# Preclinical characterization of EGT710, an oral non-peptidomimetic reversible covalent SARS-CoV-2 main protease inhibitor

**DOI:** 10.1038/s44386-025-00030-5

**Published:** 2025-11-28

**Authors:** Stephanie A. Moquin, Suresh B. Lakshminarayana, Kamal Kumar Balavenkatraman, Hilmar Schiller, Allison Claas, Barun Bhhatarai, Ioannis Loisios-Konstantinidis, Katarina Vulic, Chaitanya Kurhade, Birte K. Kalveram, John Yun-Chung Chen, Jing Zou, Xuping Xie, Laura Tandeske, Dustin Dovala, Elizabeth Ornelas, Mark S. Knapp, Daniel Fuller, Zachary Nguyen, David T. Barkan, Lidiya Bebrevska, S. Kirk Wright, Scott A. Busby, Johanne Blais, Pei-Yong Shi, Suzanne Gaudet, Renee Bergeron, Hannah Yu, Julia Zack, Christopher Sarko, Feng Gu, James E. Bradner, John A. Tallarico, Thierry T. Diagana, Julien P. N. Papillon

**Affiliations:** 1 Biomedical Research, Novartis, Emeryville, CA USA; 2 Biomedical Research, Novartis, Basel, Switzerland; 3 Biomedical Research, Cambridge, MA USA; 4 Development, Novartis, Cambridge, MA USA; 5 University of Texas Medical Branch, Galveston, TX USA; 6 Biomedical Research, Novartis, East Hanover, NJ USA; 7 Present Address: Frontier Medicines, Boston, MA USA; 8 Present Address: Pfizer, Pearl River, NY USA; 9 Present Address: Glaxo Smith Kline, Rockville, MD USA; 10 Present Address: Amgen, Thousand Oaks, CA USA; 11 Present Address: Sanofi, Cambridge, MA USA

**Keywords:** Biotechnology, Diseases, Drug discovery, Microbiology

## Abstract

EGT710 is an orally bioavailable non-peptidomimetic reversible covalent coronavirus main protease (Mpro) inhibitor with low nM cellular activity against SARS-CoV-2. Twice daily dosing of 10 mg/kg of EGT710 decreased lung viral load in a mouse model of SARS-CoV-2 infection to below the limit of detection. Resistance selection resulted in the emergence of several Mpro mutations, with recombinant viruses containing L50F + E166A substitutions showing the largest shift in potency. Development of a viral kinetics model using viremia data from clinical trials, along with a human physiologically based pharmacokinetic model, predicted efficacy in humans with once daily oral doses of >360 mg. EGT710 displays favorable pharmacokinetic properties and an acceptable in vitro and in vivo safety profile, with human exposures at the recommended clinical dose of 600 mg predicted to be below the no adverse effect level in preclinical toxicology studies. Together, EGT710 has a promising preclinical profile and has completed a Phase I study.

## Introduction

SARS-CoV-2 is the third highly pathogenic coronavirus to emerge in the last 20 years. Although effective vaccines have been developed at unprecedented speed, there remains a need to develop antivirals that are not only effective against SARS-CoV-2, but also emerging coronaviruses. Early in the SARS-CoV-2 pandemic, drug repurposing and discovery efforts quickly focused on the most well conserved and well-validated targets, the RNA-dependent RNA polymerase and the main protease (Mpro, or 3CL-protease). Mpro is a cysteine protease that cleaves the large viral polyproteins pp1a and pp1ab at eleven sites to produce twelve individual non-structural proteins that subsequently assemble to form the replication complex. Mpro is an ideal drug target because it is essential for viral replication, highly conserved across different coronaviruses, and has no known homologs in the human genome.

For antivirals, a major goal is a once daily oral drug. Paxlovid^1^ was the first fully FDA-approved oral coronavirus inhibitor. It is taken twice daily and combines nirmatrelvir (an Mpro inhibitor) with ritonavir (a CYP3A4 inhibitor and pharmacokinetic booster). However, ritonavir can limit Paxlovid’s use because of significant drug-drug interactions. Xocova/ensitrelvir^2^, an oral non-peptidomimetic Mpro inhibitor that does not require the use of a pharmacokinetic booster is approved in Japan for treatment and was recently approved by the FDA for post-exposure prophylaxis. Although several other oral Mpro inhibitors that do not require co-administration of a pharmacokinetic booster have progressed to Phase II studies, PBI-0451^3^ and EDP-235^4^ have discontinued development, S-892216^5^ is in Phase II, and ibuzatrelvir^6^ is in Phase III. Thus, oral coronavirus inhibitors that do not require co-administration of a pharmacokinetic booster are still needed.

In our drug discovery efforts, we focused on non-peptidomimetic Mpro inhibitors with the goal of developing an orally bioavailable drug with once daily dosing. The medicinal chemistry efforts that led to the discovery of EGT710, a reversible covalent, non-peptidomimetic Mpro inhibitor are described in ref. ^7^. Here, we describe the preclinical characterization of EGT710, including potency across different cellular assays, resistance development studies, efficacy in animal models, animal pharmacokinetics along with absorption, distribution, metabolism and excretion (ADME) properties, in vitro drug-drug interaction and safety profiling, as well as human dose predictions. Overall, EGT710 displays an acceptable pre-clinical profile and has completed a Phase I safety study (NL-OMON53410^8^).

## Results

### Identification of coronavirus Mpro inhibitor EGT710

**Fig. 1:**
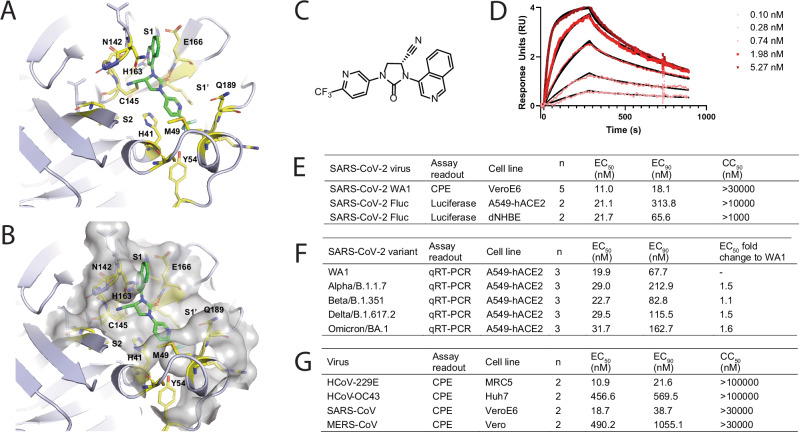
EGT710 is a non-peptidomimetic reversible covalent Mpro inhibitor with potent cellular activity against SARS-CoV-2 and other coronaviruses. **A**, **B** Co-crystal structure of EGT710 bound to SARS-CoV-2 Mpro (PDB 9OIX) with **A** surface representation and **B** non-surface representation. **C** Chemical structure of EGT710. **D** Surface plasmon resonance sensorgrams of EGT710 interaction with his-tagged SARS-CoV-2 Mpro. **E**–**G** Cellular activity of EGT710 against **E** SARS-CoV-2 in different cell lines, **F** different SARS-CoV-2 variants, and **G** other alpha- and beta- coronaviruses. Figures generated using PyMol and GraphPad Prism software.

The screening and medicinal chemistry efforts that led to the discovery of EGT710 (Fig. 1C) are described elsewhere^7^. Briefly, a virtual screen was performed, and ~5200 compounds were selected for screening along with a library of ~42,000 covalent compounds in an Mpro cleavage assay using an Rh110-tagged peptide substrate. Hits were screened in dose response and validated using orthogonal assays. Following extensive structure-activity relationship (SAR) optimization, including scaffold morphing and the addition of an electrophilic handle, EGT710 was identified. X-ray crystal structures of EGT710 in complex with SARS-CoV-2 Mpro revealed the electrophilic nitrile forming a covalent bond with Cys145 (Fig. 1A, B). To investigate whether EGT710 is a reversible or irreversible covalent inhibitor, surface plasmon resonance was used. EGT710 bound SARS-CoV-2 Mpro protein with an on-rate (ka1) of 1.02 E + 07 M^−1^s^-1^ and formed an encounter complex with an affinity (Ki) of 1.93 nM. EGT710 exhibited an off-rate (kd1) of 1.97 E + 02 s^-1^, suggesting reversible binding (Fig. 1D).

### EGT710 is a potent inhibitor of coronavirus Mpro in biochemical and cellular assays

We examined the potency of EGT710 against SARS-CoV-2 and other coronaviruses in biochemical and cellular assays. In biochemical cleavage assays, EGT710 was most potent against SARS-CoV-2 Mpro, but demonstrated activity against 8 Mpro enzymes across the four coronavirus genera, with IC_50_ values ranging from <2.5 nM to 297 nM (Table S1). EGT710 displayed an EC_50_ value of 11 nM against SARS-CoV-2 in a VeroE6 cytopathic effect (CPE) assay and displayed comparable potency (21.1 and 21.7 nM) in more relevant cell lines such as A549-hACE2 (human lung) cells and primary differentiated normal human bronchial epithelial cells (dNHBE) grown in an air-liquid interface, also known as human airway epithelial (HAE) cells (Fig. 1E). EGT710 also maintained potency against different SARS-CoV-2 variants, with EC_50_ values ranging from 22.7 nM to 31.7 nM, and <1.6-fold shifts from SARS-CoV-2 wildtype (WA1) (Fig. 1F). To determine the breadth of EGT710 across coronaviruses, we also determined cellular potency against other human coronaviruses. EGT710 had similarly potent activity against SARS-CoV and HCoV-229E, with EC_50_ values of 18.7 and 10.9 nM, respectively, but reduced activity against HCoV-OC43 and MERS-CoV with EC_50_ values of 456.6 nM and 490.2 nM, respectively (Fig. 1G). Structural studies of EGT710 bound to Mpro from HCoV-OC43 and MERS-CoV did not reveal large differences in binding that could explain the differences in potency (Fig. S1).

### Resistance profile of EGT710

**Fig. 2:**
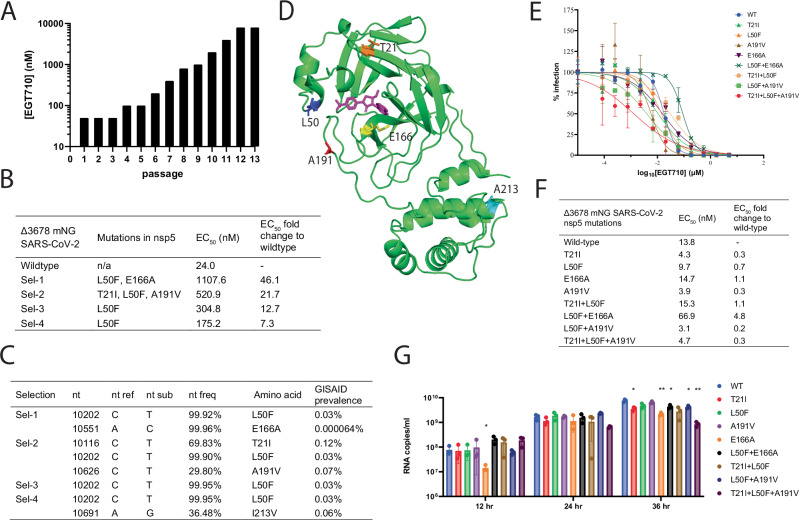
In vitro resistance selection of EGT710 and characterization of selected viruses. **A** Concentration of EGT710 in serial passaging experiments performed with Δ3678 mNG SARS-CoV-2 in VeroE6-TMPRSS2 cells for selections 1, 2, and 4, reaching a maximum concentration of 8 µM. Selection 3 reached a maximum concentration of 4 µM. **B** Susceptibility of the selected virus population to EGT710 after 13 passages determined in VeroE6-TMPRSS2 cells using qRT-PCR at 24 h post-infection. Geometric mean EC_50_ values from n = 2 independent experiments. **C** Prevalence of nucleic acid substitutions present in the nsp5 gene identified by next generation sequencing (NGS) at passage 13, and prevalence in GISAID sequences. Nt, nucleotide; nt ref, nucleotide reference; nt sub, nucleotide substitution; nt freq, nucleotide frequency by NGS **D** Location of amino acid substitutions relative to the EGT710 binding site. **E**, **F** Susceptibility of recombinant Δ3678 mNG SARS-CoV-2 containing nsp5 mutations to EGT710 determined in VeroE6-TMPRSS2 cells using high-content imaging at 24 h post-infection: **E** representative concentration-response curves from a single experiment and **F** geometric mean EC_50_ values from n = 2 independent experiments. **G** Fitness of recombinant Δ3678 mNG SARS-CoV-2 containing nsp5 mutations. VeroE6-TMPRSS2 cells were infected with 0.1 MOI of recombinant viruses, and qRT-PCR was used to quantify RNA copies/mL in the supernatant at 12, 24, and 36 h post-infection. Bars are mean value from n = 3 experiments ± standard deviation; *, p < 0.05; **p < 0.005. Figures generated using PyMol and GraphPad Prism software.

To determine the resistance profile of EGT710, we performed four independent serial passage selections (Se1 1 - Sel 4) with increasing concentrations of EGT710 using the attenuated Δ3678 mNG SARS-CoV-2^9^ in VeroE6-TMPRSS2 cells. After 13 passages, the concentration of EGT710 tolerated by the virus was 4-8 µM, or approximately 200- to 400-fold over the EC_50_ (Fig. 2A), and the selected viral populations showed between 7.3- and 46.1-fold decrease in potency when compared to wildtype virus (Fig. 2B). Next-generation sequencing of the viral populations identified mutations in the nsp5 gene resulting in Mpro amino acid substitutions T21I, L50F, E166A, A191V, and I213V in different selections (Fig. 2C). Of these, L50F and E166A were the only two substitutions present in >99% of sequences at passage 13 (P13) in any of the selected viral populations (Fig. 2C). The five substitutions appear in different regions of the Mpro protein, with only E166A appearing near the binding site of EGT710 (Fig. 2D). Because I213V was present at low percentage frequency at P13 and is located far from the active site, it was not further investigated. Additional mutations appeared in other areas of the viral genome (Table S2), but these were not further investigated in this study.

To investigate the contribution of each individual mutation to resistance, recombinant viruses containing single, double, and triple mutations were generated in the attenuated Δ3678 mNG SARS-CoV-2 backbone, and recombinant viruses were recovered. Next, we evaluated the potency of EGT710 against the recombinant viruses in VeroE6-TMPRSS2 cells. The EC_50_ value against the wildtype virus was 13.8 nM, while EC_50_ values against recombinant viruses ranged from 3.1 nM to 66.9 nM. The virus containing L50F + E166A substitutions showed the largest shift in potency, with an EC_50_ value of 66.9 nM and 4.8-fold shift in potency compared to wildtype (Fig. 2E, F). We then examined the fitness of the recombinant viruses by measuring viral genome copies over time in VeroE6-TMPRSS2 cells infected with equivalent multiplicity of infection (MOI). The virus with the single E166A amino acid substitution had significantly lower RNA copies than the other mutant viruses at 12 hours, and five viruses had significantly lower RNA copies than wildtype at 36 hours: T21I, E166A, L50F + E166A, L50F + A191V, and T21I + L50F + A191V (Fig. 2G).

To determine the prevalence of these mutations in clinical isolates, we examined 15,593,310 sequences available from the Global Initiative on Sharing Avian Influenza Data (GISAID, gisaid.org) database as of January 6, 2025. All mutations were present at <0.12% prevalence, with E166A appearing in only 10 total sequences (0.000064%) (Fig. 2B).

### EGT710 is efficacious in a SARS-CoV-2 mouse model

**Fig. 3:**
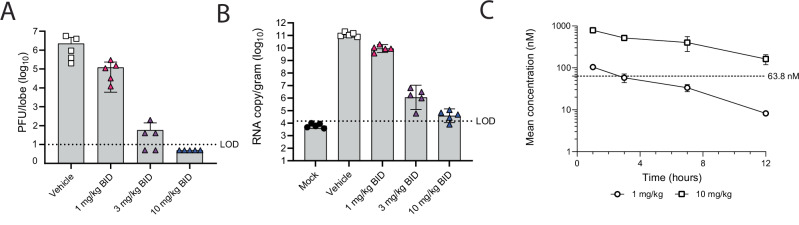
Efficacy of EGT710 in a mouse model of SARS-CoV-2 infection. Balb/c mice were intranasally infected with 10^4^ PFU mouse-adapted SARS-CoV-2 strain CMA4. Oral dosing was initiated immediately after infection, and mice were dosed twice daily (BID) for 2 days. Two days after infection, right lung lobes were collected for plaque assay or qRT-PCR. Viral titers **A** and RNA copies **B**) in mouse lungs two days post-infection after treatment with EGT710. **C** Unbound concentration of EGT710 in uninfected Balb/c mice following a single oral dose of either 1 mg/kg or 10 mg/kg. Dotted line indicates the unbound EC_90_ = 63.8 nM (EC_90,assay_ = 65.6 nM, adjusted based on 2.82 % protein binding of EGT710 in the dNHBE media (Table 1)). Figures generated using GraphPad Prism software.

We observed that EGT710 has a favorable pharmacokinetic (PK) profile in rats, with a single oral 10 mg/kg dose maintaining the C_trough_ above the unbound EC_90_ in dNHBE cells (63.8 nM) for ~23 h^7^. Thus, we tested the efficacy of EGT710 in a mouse SARS-CoV-2 infection model using the mouse-adapted SARS-CoV-2 strain CMA4. This virus contains two mutations in the spike protein that allow for binding to mouse ACE2 and infection of wildtype mice. Balb/c mice were infected with 10^4^ PFU of SARS-CoV-2 CMA4 and orally dosed twice a day (BID) with EGT710 for two days. Two days after infection, right lung lobes were collected for plaque assay or qRT-PCR. Treatments with 1, 3 or 10 mg/kg BID EGT710 resulted in a 1.27, 4.59 or >5 log decrease in lung viral titer (PFU/lobe) and a 1.08, 4.71 or >5 log decrease in lung RNA copies/mL (Fig. 3A,B). In parallel, we examined the pharmacokinetics of EGT710 in uninfected mice following a single oral dose. Mice in the 1 and 10 mg/kg group maintained unbound C_12h_ concentration approximately 0.13- and 2.5-fold the unbound EC_90_ value of EGT710 in dNHBE cells, respectively (Fig. 3C).

### EGT710 has favorable in vitro ADME properties

Some of the ADME properties of EGT710 including low clearance in rat and human microsomes as well as good oral bioavailability in rats at low doses ( <10 mg/kg) are reported elsewhere^7^; here we report more extensive profiling across different species and in additional assays to prepare for clinical studies. EGT710 displayed high passive permeability and low metabolic turnover in microsomes and hepatocytes across all tested species (Table 1). In vitro plasma protein binding was moderate in all tested species (Table 1), and no concentration-dependence was found between assays run at 1 and 10 µM. EGT710 distributed roughly equally between plasma and blood cells in all tested species, except in rabbit, where a higher association with blood cells was observed (Cb/Cp = 1.49) (Table 1). Blood-to-plasma distribution was not concentration-dependent between 10 and 10,000 ng/mL.

**Table 1:** In vitro ADME properties of EGT710

Property	Value
P_app_ MDCK-LE (x10^-6^ cm s^−1^)	21.4
MDCK-MDR1 (x10^-6^ cm s^-1^) P_app_A-B / P_app_B-A / ER	7.4 / 13.0 / 1.8
Cl_int_, microsomes (µL/min/mg) (M/Rt/D/H)	<10 / < 10 / 13.3 / < 10
Cl_int_, hepatocytes (µL/min/million cells) (M/Rt/D/H)	5.2 / < 4 / < 4 / < 4
Plasma protein binding (%) (M/Rt/Rb/D/H)^a^	81.9 / 86.1 / 74.3 / 76.1 / 88.2
Binding to dNHBE media (%)	2.82
Cb/Cp (M/Rt/Rb/D/H)^b^	0.83 / 1.10 / 1.49 / 0.92 / 1.21

*P*_app_ apparent permeability coefficient, *MDCK-LE* Madin-Darby canine kidney low efflux cells, *MDCK-MDR1* Madin-Darby canine kidney cells expressing multidrug resistance 1, *CL*_int_ intrinsic clearance, *M/Rt/Rb/D/H* mouse/rat/rabbit/dog/human, *Cb/Cp* blood to plasma ratio.

^a^PPB values from discovery-stage rat assay are reported in ref. ^7^ here, we report PPB values from IND-enabling assays for all species. Mean values for experiments performed at 1 and 10 µM are reported.

^b^Mean values for experiments performed at 10, 100, 1000, and 10,000 ng/mL are reported.

Metabolism of EGT710 across species was assessed in vitro. In hepatocytes of mouse, rat, rabbit, dog, and human, EGT710 was metabolized to eleven metabolites in total. The two main reaction pathways were non-enzymatic conjugation with cysteine including downstream reactions (conjugation with glutathione, oxidation, hydroxylation) and formation of an oxidative metabolite. No human-specific metabolites were observed (Fig. S2), suggesting that any metabolites formed in humans would be represented in animal toxicology studies.

### EGT710 has good oral bioavailability and low clearance in vivo

To prepare for toxicology studies and human dose projections, we assessed the pharmacokinetics of EGT710 in mouse, rat and dog. Following single intravenous (i.v.) administration, EGT710 showed low blood clearance ( <10% of hepatic blood flow in mouse and rats, <20% of hepatic blood flow in dogs) and moderate-to-high volume of distribution resulting in moderate-to-long half-life (T_1/2_) across species (Table 2). Following single oral (p.o.) administration, EGT710 was rapidly absorbed with median T_max_ between 1.0 and 3.0 h post-dose. Oral bioavailability was moderate in mouse (39.3%) and high in rats (100%) and dogs (84.3%) (Table 2).

**Table 2:** Pharmacokinetic parameters of EGT710 following single intravenous or oral dosing in mouse, rat and dog

	Oral PK parameters					Intravenous PK parameters				
Species	Dose (mg/kg)	Cmax (ng/mL)	Tmax mean (h)	AUC_inf_ (µg x h/mL)	F (%)	Dose (mg/kg)	Vss (L/kg)	CL (mL/min/kg)	T1/2 (h)	AUC_inf_ (µg x h/mL)
Mouse	30	5443	1.0	36.38	39.3	1.0	1.30	5.51	2.64	3.25
Rat	10^a^	2422	2.5	34.61	>100	1.0	2.01	4.86	5.67	3.43
	100^a^	14795	1.0	421.63	>100					
Dog	10	2066	2.3	27.33	84.3	1.0	3.67	5.18	8.38	3.23

*Cmax* maximum concentration achieved; Tmax time of peak concentration, *AUC*_inf_ area under the curve from time 0 to infinity, *F* absolute oral bioavailability, *Vss* volume of distribution at steady state, *CL* total systemic clearance, *T*_1/2_ terminal elimination half-life.

^a^data presented in ref. ^7^ was converted to ng/ml using the molecular weight of 383.3 g/mol.

### EGT710 has low to moderate potential for drug-drug interactions

The potential of EGT710 as a perpetrator or a victim of drug-drug interactions (DDIs) was assessed in vitro. EGT710 was a reversible inhibitor of CYP2C8 (K_i,u_ = 50.5 µM) and CYP3A4 (K_i,u_ = 33.3 µM) and an inducer of CYP1A2, CYP2B6, CYP2C8, CYP2C9 and CYP3A4 (Table S3). Physiologically-Based Pharmacokinetic (PBPK) modeling predicted a weak induction of CYP2C8 and moderate induction of CYP3A4 at therapeutic exposures (see Supplementary Note). In vitro, EGT710 inhibited several uptake and efflux transporters (Table S3). Based on static DDI assessment, EGT710 may weakly inhibit renal uptake transporters MATE1, MATE2-K and OAT3, and intestinal efflux transporter MDR1 at clinical doses. EGT710 is not expected to be a victim drug of transporter-mediated DDIs because of its high passive permeability. CYP-mediated oxidative metabolism of EGT710 was mainly via CYP3A4 with potential contribution by extrahepatic CYP1A1.

### EGT710 is predicted to have good pharmacokinetic properties in human

Using the PK data obtained from preclinical species, EGT710 human PK was predicted using an average of relevant methods for allometric scaling from mouse, rat, and dog and the Wajima approach to predict a CL of 2.2 mL/min/kg, a V_ss_ of 2.32 L/kg, and a half-life of ~14 h. Combining disposition parameters with physicochemical, solubility, and permeability data, the oral bioavailability and absorption kinetics in human were predicted using the ACAT model in GastroPlus^TM^ (see Supplementary Note). The EGT710 bioavailability is predicted to be dose-dependent (range of ~55–76% for doses <700 mg). The projected human CL, V_ss_, and T_1/2_ appeared adequate to achieve pharmacologically active exposures using an oral QD dosing regimen.

### Doses greater than 360 mg once daily (QD) EGT710 are predicted to be efficacious in humans, with 600 mg QD recommended for evaluation in the clinic

Although viral load reduction has been reported for various COVID-19 therapeutics, at the time of this work in 2021, there was insufficient data to establish a model relating viral load reduction to clinical response. Challenges in predicting clinical efficacy include the potential for mechanism-of-action-specific relationships between viral load reduction and clinical response which may be further confounded by different circulating SARS-CoV-2 variants during different clinical trials. Of note, inhibitors of the RNA-dependent RNA polymerase (RdRP) such as remdesivir and AT-527 have shown clinical response in the absence of viral load reduction^10–12^. As such, the conservative efficacious dose prediction strategy employed was to benchmark EGT710 viral load predictions to be equivalent to predictions for the clinical regimen of Mpro inhibitor nirmatrelvir+ritonavir that resulted in clinical response^13^, thus assuming that equivalent viral load reduction by Mpro inhibitors will translate into equivalent clinical response.

To this end, a viral kinetics model was built to describe population variability in natural disease progression of SARS-CoV-2. First, to generate a model specific to the delta variant, a one-state immune model built based on the wildtype SARS-CoV-2 variant (data from ref. ^14^, model from ref. ^15^) was re-parameterized with data from a patient population predominantly infected with the delta variant (ref. ^16^, placebo group). Next, the anti-replicative mechanism of Mpro inhibitors was incorporated into the untreated base model by including information about predicted human PK, in vitro anti-viral potency, and plasma protein binding. To validate the model, model predictions were made for Mpro inhibitors nirmatrelvir+ritonavir and ensitrelvir. Upon comparison to reported data for clinical response of viral load change from baseline, the delta variant model under untreated conditions was validated and response was largely predicted within model variability, albeit with some systematic over-prediction (Fig S3).

**Fig. 4:**
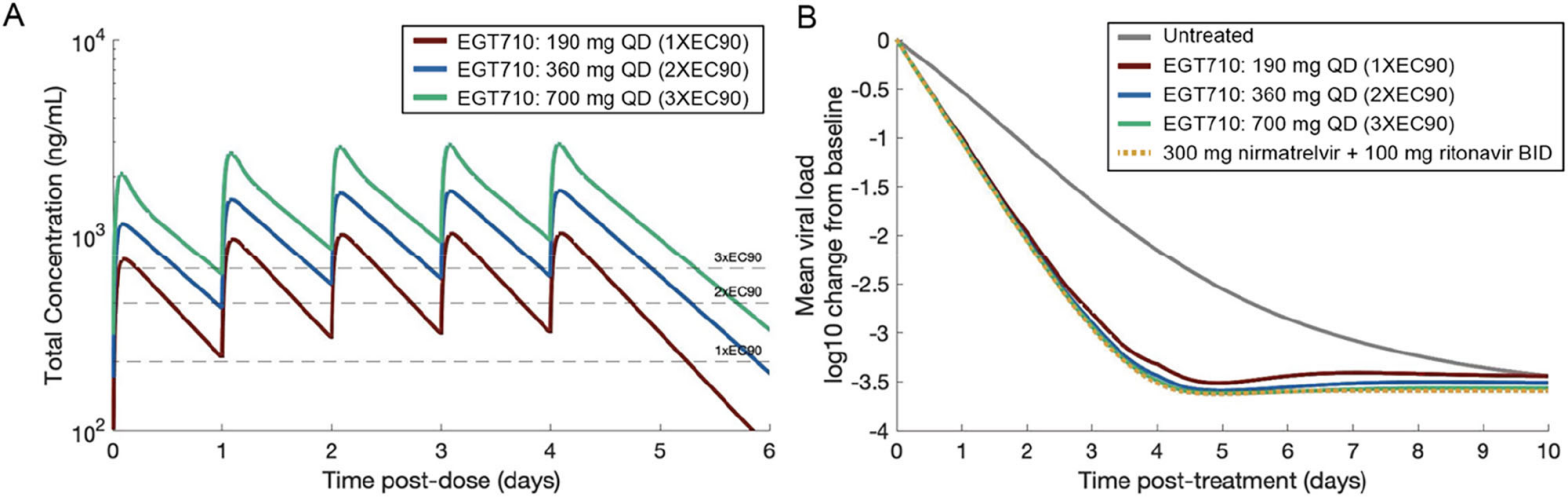
Viral kinetics model predicts efficacious dose of EGT710 to be >360 mg QD. **A** Compartmental model prediction of total EGT710 PK (fasted state) relative to *EC90*_*total*_ value. **B** Mean viral kinetics simulations for EGT710 (green, blue, and red lines) compared to untreated (gray line) and 300 mg nirmatrelvir + 100 mg ritonavir BID (yellow line). Figures generated using MATLAB software.

Based on the confidence in the viral kinetics PK/PD model for Mpro inhibitor treatment built from the validation against nirmatrelvir+ritonavir and ensitrelvir data, model simulations were performed for various EGT710 regimens. Model simulations for EGT710 compared to nirmatrelvir+ritonavir and ensitrelvir simulations support doses targeting 24 h Cmin > 2x total EC_90_ value to achieve comparable and near saturating mean anti-viral response (Fig. 4). Combining the viral kinetics model results with the predicted human EGT710 PK (fasted vs fed states) and their associated uncertainties, doses greater than 360 mg QD are predicted to result in a clinical viral load reduction comparable to those generating favorable clinical responses with other Mpro inhibitors (Fig. 4, Table S4). Taken together with uncertainties in the modeling predictions, large population variability in viral load trajectories, variability in the severity of disease at onset of therapy, and feasibility and formulation considerations, a dose of 600 mg QD was recommended for clinical evaluation for efficacy.

### EGT710 has an acceptable preclinical safety profile

The preclinical safety profile of EGT710 was investigated both in vitro and in vivo. In vitro, EGT710 displayed no significant inhibition or stimulation against 16 human peptidases (Table S5), and EGT710-related inhibition was found only on 2/103 targets (ion-channels, transporters, enzymes, GPCRs, etc) (Table S6), but was not considered to be clinically relevant. EGT710 was not phototoxic in a 3T3 NRU assay and was not genotoxic in an Ames assay or micronucleus assay in human TK6 cells. EGT710 inhibited hERG channel activity in vitro with an IC_50_ = 143 µM. The effect of EGT710 on the cardiovascular system was further investigated in vivo (see below).

To examine the in vivo safety profile of EGT710, several studies were performed. To assess the effect of EGT710 on the central nervous system and respiratory function, a good laboratory practice (GLP) study in male Wistar Han rats was performed with a single oral administration of 250 mg/kg EGT710. No treatment-related effects were noted up to 24 hours following dosing. To assess the effect of EGT710 on the cardiovascular system, a telemetry GLP study with continuous monitoring of cardiovascular parameters was performed in male dogs following single oral administration up to a dose of 200 mg/kg. No evidence of cardiac arrhythmias was noted. To assess overall toxicity, two-week GLP repeat oral dosing toxicity studies were conducted. In rats and dogs, EGT710 plasma exposure increased across increasing dose levels but was generally less than dose proportional in both sexes. In rats dosed for two weeks with oral doses between 10 and 250 mg/kg/day, exposure increased roughly proportionally up to 25 mg/kg/day and under-proportionally from 25 to 250 mg/kg/day. There was no accumulation of EGT710 after multiple administration and no sex difference were noted. In dogs dosed for two weeks with doses between 30 and 300 mg/kg/day, exposure increased slightly under-proportionally without accumulation or sex differences. The no-observed-adverse-effect-level (NOAEL) was 250 mg/kg in rats and 30 mg/kg in dogs. The exposures at the NOAEL dose in rats and dogs are at least 7- and 3-fold higher than the predicted human exposure at the recommended clinical dose of 600 mg QD, respectively. The effect of EGT710 on reproductive toxicity was also examined. In a rat fertility and early embryonic development (FEED) study and a rat embryo-fetal development (EFD) study, the NOAEL was 300 mg/kg/day, the highest tested dose in both these studies. In the rabbit EFD study, EGT710 was teratogenic and induced vertebral skeletal malformations at 100 mg/kg/day. The NOAEL for embryo-fetal development in rabbits was 50 mg/kg/day. The exposure at this dose of 50 mg/kg/day was slightly below the predicted human exposure at the recommended clinical dose of 600 mg QD.

## Discussion

Here we describe the preclinical characterization of EGT710, an orally bioavailable non-peptidomimetic reversible covalent inhibitor of coronavirus Mpro. Our drug discovery efforts were focused on non-peptidomimetic compounds, as peptidomimetic compounds often display poor physicochemical properties, low stability, and low oral bioavailability. This enabled the discovery of EGT710, which displays good physicochemical properties with low clearance and good oral bioavailability, with once-daily doses predicted to be efficacious in humans without the use of a pharmacokinetic booster such as ritonavir.

EGT710 displays low nM potency against SARS-CoV-2 and is similarly efficacious against all tested variants of SARS-CoV-2. As all omicron sublineages identified to date contain only one amino acid substitution in Mpro (P132H), we expect that the activity of EGT710 against the other omicron sublineages is similar to the activity of EGT710 measured against BA.1 used in this study. While EGT710 is similarly potent against SARS-CoV-2, SARS-CoV, and 229E in cellular assays, it displays an ~25-fold shift in potency against HCoV-OC43 and MERS-CoV. For a novel emerging coronavirus, sensitivity to EGT710 should be assessed in cell-based assays to determine whether EGT710 could reach predicted efficacious exposures in humans with appropriate safety margins. Additionally, some analogs of EGT710 display improved spectrum of activity across coronaviruses^7^; these analogs could also be assessed for potency against a novel emerging coronavirus.

To understand the resistance profile of EGT710, we performed in vitro selection experiments, resulting in the appearance of Mpro amino acid substitutions T21I, L50F, E166A, A191V, and I213V. In vitro resistance selection experiments have been performed for nirmatrelvir^17–19^, which also targets the same binding pocket as EGT710. In these studies, substitutions of T21, L50, A191, and E166 were also observed. Two studies identified E166V mutations, with recombinant viruses showing 34- to 130-fold shifts in nirmatrelvir potency^17,18^. T21I and L50F appear in all three studies, and while each mutation alone does not result in large shifts in potency, they have been proposed to restore the fitness of less-fit but more highly resistant viruses such as E166V^18^. Similarly, we observed the largest shift in potency for EGT710 in the L50F + E166A mutant (4.8-fold), though it was substantially lower than the shift in potency observed for nirmatrelvir against L50F + E166V viruses. We also observed that the E166A mutant appeared to be less fit (i.e., had significantly lower RNA copies than wild-type virus at 12 h), while the L50F + E166A virus has similar copies as wildtype, supporting the theory that L50F may restore fitness of E166 mutant viruses. Interestingly, shifts in potency against the recombinant viruses were lower than against the EGT710-selected viral populations, which could indicate that other mutations outside of Mpro may be playing a role in resistance, or it could simply be because the selected viral populations are a mixture of many different viruses. While substitutions in E166 are rare in SARS-CoV-2 sequences from patients, as observed by this group and others^19^, their prevalence should continue to be monitored as Paxlovid continues to be used to treat patients with SARS-CoV-2 infections, especially in immunocompromised patients, where resistance often first emerges. Additionally, in vitro studies examining the potency of EGT710 against nirmatrelvir-resistant viruses would provide insight into whether EGT710 could be used against any nirmatrelvir-resistant viruses that emerge in the clinic.

Overall, the non-peptidomimetic nature of EGT710 resulted in good in vitro and in vivo pharmacokinetic properties in rodents and dog, such as low clearance, good permeability, and good oral bioavailability. These properties resulted in efficacy in a mouse model of SARS-CoV-2 infection. In humans, EGT710 is predicted to reach the efficacious exposure of >2x EC_90_, the threshold informed by our viral kinetics model, with an oral dose of >360 mg QD, with the recommended clinical dose of 600 mg QD maintaining exposure multiples of 7- and 3-fold above the NOAEL in rat and dog, respectively. Based on the combination of in vitro results and modeling, EGT710 is predicted to have a weak to moderate potential for perpetrator drug-drug interactions. Thus, EGT710 would have a reduced potential for perpetrator drug-drug interactions when compared to compounds that are co-dosed with ritonavir. However, because EGT710 metabolism is mediated mainly by CYP3A4, the potential of EGT710 to be a victim of DDI should be evaluated in a clinical study using CYP inhibitors.

With this favorable preclinical package, we initiated and completed Phase I clinical studies for EGT710 to assess safety and pharmacokinetics in healthy human volunteers (NL-OMON53410^8^). With Phase I studies complete, EGT710 is ready to be deployed in a Phase II efficacy study against SARS-CoV-2. Of consideration for the future development of EGT710 is that reproductive toxicity was observed for EGT710 in rabbit EFD studies, with exposures at the NOAEL slightly below the predicted efficacious exposure at the recommended clinical dose of 600 mg QD. Therefore, in any future human studies, EGT710 should be administered together with effective contraceptive measures. Additionally, as seen in later-stage pandemic clinical trials, demonstrating efficacy of SARS-CoV-2 antivirals has become increasingly difficult because a growing proportion of the population is vaccinated or has prior immunity, resulting in milder disease, faster viral clearance, and shorter symptom duration^20–22^. Nevertheless, in the event of a novel coronavirus emergence, if EGT710 is sufficiently potent to be administered at predicted efficacious doses with appropriate exposure margins, EGT710 could be immediately deployed in Phase II efficacy studies against a novel coronavirus.

## Methods

### Viral assays

SARS-CoV-2 CPE assay (72 h) was performed in VeroE6 cells, 229E CPE assay (72 h) was performed in MRC5 cells, OC43 CPE assay (168 h) was performed in Huh7 cells, SARS-CoV-1 CPE assay (72 h) was performed in VeroE6 cells, and MERS CPE assay (96 h) was performed in Vero81 cells. Potency against SARS-CoV-2 in dNHBE cells was measured in a 72 h assay using SARS-CoV-2 firefly luciferase reporter virus and luciferase as a readout. Potency against SARS-CoV-2 in A549-hACE2 cells was measured in a 48 h assay using SARS-CoV-2 firefly luciferase reporter virus and luciferase as a readout. Potency against SARS-CoV-2 variants was measured in a 48 h assay in A549-hACE2 using qRT-PCR as a readout. Additional details are in the Supplementary Note.

### Resistance selection

VeroE6-TMPRSS2 cells were collected and resuspended in assay media (DMEM supplemented with 2% FBS and 1% penicillin-streptomycin. A total of 2.0×10^5^ cells suspended in 0.5 mL assay media were seeded in each well of a Nunc™ Cell-culture treated 24-well plate, and plates were incubated overnight at 37 °C in 5% CO_2_. For each passage, a 200 µL aliquot of harvested virus from previous passage was added to each well of the 24-well plate seeded with VeroE6-TMPRSS2 cells and plates were incubated at 37 °C for 1 h. Following the infection period, the supernatant containing free virus was removed and replaced with 500 µL of assay medium supplemented with increasing concentrations of EGT710. When more than 90% of cells were green and/or >30% CPE was observed, supernatants were harvested and stored at -80°C. Typically, DMSO controls were harvested on day 2 post-infection while EGT710 selections were harvested on day 5–6 post-infection. For RNA extraction from P13 viral populations, a 100 µL aliquot of P13 supernatants from each of the four independent serial passage selections was collected and mixed with 500 µL of TRIzol™ LS (Thermo Fisher Scientific), RNAs were extracted by the Direct-zol™ RNA Miniprep Plus kit (Zymo Research) and eluted in 50 µL Rnase-free water. Viral RNA sequencing libraries were prepared using the NEBNext Ultra II RNA library prep kits (NEB) following the manufacturer’s non-directional protocol. Methods for sequencing analysis can be found in the Supplementary Note.

### Evaluation of potency against passaged Δ3678 mNG SARS-CoV-2

2.5 × 10^4^ cells suspended in 50 μL assay media were seeded in each well of a 96-well white opaque flat-bottom plate and plates were incubated overnight at 37 °C in 5% CO_2_. On the day of the assay, wildtype Δ3678 mNG SARS-CoV-2 and P13 mutants were diluted to a concentration of 5 × 10^4^ plaque forming units (PFU)/mL using assay media, and 200 µL of diluted virus was added to 2 µL of serially diluted compound. Fifty μL of the compound-virus mixture from a single well were added to VeroE6-TMPRSS2 cells, resulting in a multiplicity of infection (MOI) of approximately 0.1. Concentrations were tested in triplicates. Plates were mixed on an IKA MS 3 digital orbital shaker for 90 s at 500 rpm. After 1 h infection, the inoculum was replaced by 100 µL of fresh medium assay media containing 200× of serially diluted compound or DMSO. The plates were incubated at 37°C. At 24 hours post-infection, supernatants were removed, and cells were lysed with 100 µL of TRIzol™ LS (Thermo Fisher Scientific). Cell lysates (100 µL) were transfer to fresh tubes containing 300 µL of TRIzol™ LS. RNAs were extracted by the Direct-zol™ 96 Magbead kit (Zymo Research) using the KingFisher™ Flex system (Thermo Fisher Scientific) and eluted in 50 µL Rnase-free water. qRT-PCR was performed to quantify the viral RNA copies.

### Imaging assay against Δ3678 mNG SARS-CoV-2 containing nsp5 mutations

2.5 × 10^4^ VeroE6-TMPRSS2 cells suspended in 50 μL assay media (DMEM with 2% FBS 1% Penicillin-Streptomycin) were seeded in each well of a 96-well in black flat-bottom (Grenier Bio-One), and plates were incubated overnight at 37 °C in 5% CO_2_. On the day of the assay, 200 µL of diluted wildtype or mutant Δ3678 mNG SARS-CoV-2 viruses were added to 2 µL of serially diluted compound. 50 μL of the compound-virus mixture from single wells were added to VeroE6-TMPRSS2 cells. Concentrations were tested in triplicates. Plates were mixed on an IKA MS 3 digital orbital shaker (IKA Works, Inc.) for 90 s at 500 rpm. After 1 h infection, the inoculum was replaced by 100 µL of fresh medium assay media containing 200X of serially diluted compound or DMSO. The plates were incubated at 37°C in 5% CO_2_ for 24 h. At 24 h post-infection, cells were counterstained with 20 µM of Hoechst 33342: stock solution was diluted 1:200 in Dulbecco’s phosphate buffered saline (DPBS) (Gibco™) and 25 µL was added to each well (final dilution 1:1000). Plates were sealed and shaken for 2 min on IKA MS 3 digital orbital shaker for at 500 rpm. Plates were further incubated at room temperature for 20 min. Images were taken on Thermo Scientific Cellinsight CX5 HCS Platform to calculate the percentage of cells infected.

### Replication kinetics of recombinant Δ3678 mNG SARS-CoV-2 viruses

VeroE6-TMPRSS2 cells were collected and resuspended in assay media (DMEM (Gibco) supplemented with 2% FBS (HyClone), and 1% Penicillin-Streptomycin (10,000 U/mL) (Gibco). A total of 3 × 10^5^ cells suspended in 1 mL assay media were seeded in each well of a 12-well plate, and plates were incubated overnight at 37 °C in 5% CO_2_. On the day of the assay, cells were infected with viruses diluted to an MOI of 0.1 in 150 µL of DMEM media supplemented with 2% FBS and plates were incubated for 1 h at 37 °C in 5% CO_2_. After infection, cells were washed twice with DPBS and supplemented with 1 mL DMEM media supplemented with 2% FBS. At various time points (12 h, 24 h, and 36 h post-infection), 100 µL of culture supernatants were collected into fresh tubes containing 300 µL of TRIzol™ LS (Thermo Fisher Scientific). One hundred microliters of fresh medium were added into each well to replenish the media volume. Each virus was tested in triplicate. All TRIzol samples were stored at -80°C until RNA isolation.

RNA was extracted with the Direct-zol™ 96 Magbead kit (Zymo Research; cat# R2102) using the KingFisher™ Flex system (Thermo Fisher Scientific) and eluted in 50 µL RNAse-free water. qRT-PCR was performed to quantify the viral RNA copies.

### Mouse model

All animal studies were reviewed and approved by the Institutional Animal Care and Use Committee of the Global Health group at Novartis and at the University of Texas Medical Branch at Galveston under protocol 2103023. Animals were randomly assigned to various treatment groups, and scientists were not blinded to treatment groups. No animals or data points were excluded from the analysis. Power calculations for animal efficacy studies were performed using data from Fig. 4 of Ku et al.^23^. Power to detect a 2-log difference in viremia with alpha = 0.1 was 66%. Female Balb/c mice (Jackson laboratories), 8-10 weeks of age were anesthetized with isoflurane and infected intranasally (IN) with 50 µL of mouse-adapted SARS-CoV-2 strain CMA4^24^ at a concentration of 2 × 10^5^ plaque forming units (PFUs)/mL in DPBS, resulting in a final inoculum of 10^4^ PFUs per mouse. Dosing began immediately after infection. Mice were dosed with 100 µL vehicle (0.5% methyl cellulose and 0.5% Tween-80) or EGT710 formulated in 0.5% methyl cellulose and 0.5% Tween-80 in water via p.o. gavage (n = 5 per group) twice daily (BID) for 2 days. All mice were monitored and weighed daily until scheduled euthanasia on Day 2. Two days after infection, mice were euthanized by CO_2_ inhalation followed by cervical dislocation or bilateral thoracotomy, and necropsied. The right lung cranial lobes were taken, weighed and immersed in a 2 mL tube containing 1 mL of DPBS. Lung samples were homogenized using the MagNA Lyster at 6000 rpm for 1 min, clarified by centrifugation at 12,000 rpm for 5 min, and collected for plaque assay on VeroE6 cells as previously described^25^. The rest of the right lung lobes were weighed and put into 2 mL tube containing 500 µL of TRIzol™ (Thermo Fisher Scientific). Lung samples placed in TRIzol™ were homogenized using the MagNA Lyser (Roche Diagnostics) with settings of 6000 rpm for 1 min. The tubes were then centrifuged at 12000 rpm for 1 min. Fresh TRIzol™ (500 µL) was added to all samples. Half volume of the samples was used to extract the total RNA by using the Direct-zol™ RNA Miniprep Kits (Zymo Research) according to the manufacturer’s instructions. RNA samples were finally eluted in 50 µL of nuclease-free water, and qRT-PCR was used to quantify RNA copies. Results were analyzed using GraphPad Prism version 9.3.1 (GraphPad Software, San Diego, CA). Statistical analysis was done using a one-way ANOVA with a multiple comparison correction using the Bonferroni method. Pharmacokinetics of EGT710 in uninfected Balb/c mice was evaluated in parallel (see pharmacokinetics section).

### qRT-PCR

Two microliters of RNA samples were used for quantitative reverse transcription polymerase chain reaction (qRT-PCR) assays in a 20-µL reaction system using the iTaq SYBR Green one-step kit (Bio-Rad) on the QuantStudio™ qRT-PCR systems with fast 96-well module (Thermo Fisher Scientific). The quantification of viral RNA was determined by a standard curve method using an RNA standard (in vitro transcribed 3839 bp RNA at the nucleotide positions from 26,044 to 29,883 of SARS-CoV-2 genome). qRT-PCR oligonucleotide sequences were 2019-nCoV_N2-F primer 5’-TTACAAACATTGGCCGCAAA-3’ and 2019-nCoV_N2-R primer 5’-GCGCGACATTCCGAAGAA-3’^26^.

### In vitro ADME, DDI, safety profiling assays

In vitro assays for MDCK-LE permeability^27^, MDCK-MDR1 efflux^28^, microsomal stability^29,30^, hepatocyte induction^31^, CYP450 phenotyping^32^, CYP450 inhibition^31^, transporter inhibition^31^ were performed as previously described. Plasma protein binding or media protein binding was determined by equilibrium dialysis for 4 h at 37 °C after spiking plasma with 1 and 10 µM EGT710. Blood to plasma distribution was measured by spiking blood with 10, 100, 1000 and 10,000 ng/mL EGT710. After incubation for 30 min at 37 °C, EGT710 concentrations were measured by LC-MS in whole blood and plasma. In vitro biotransformation in hepatocytes was assessed by incubating 10 µM EGT710 in 10^6^ hepatocytes/mL. After incubation for 6 h, samples were quenched by addition of three volumes of ice-cold acetonitrile and centrifuged for 15 min at 20,000 x g and 4 °C. Supernatants were evaporated to dryness and reconstituted in water containing 25% acetonitrile. Metabolite profiles and structural elucidation of metabolites was obtained by UPLC-MS.

In vitro activity against 16 proteases was performed by Eurofins. In vitro assays for cardiotoxicity^33^, micronucleus genotoxicity^34^, mini-AMES genotoxicity^35^, 3T3 neutral red uptake phototoxicity^36^ were performed as previously described. Assays for proteins that bear potential safety liabilities in humans were performed using recombinant protein or membrane fractions containing the protein of interest, and competitive binding assays were performed using specific radiolabeled ligands^37,38^.

### Pharmacokinetic properties and bioavailability

All procedures involving animals were reviewed and approved by the institutional animal care and use committees. No statistical methods were used to predetermine sample size, and they were chosen based on the minimum number of animals required for good data distribution and descriptive statistics. Blinding was not possible in these experiments, but animals were selected randomly for each group. In vivo pharmacokinetic studies were conducted using non-randomized C57BL/6 male mice (n = 3 per group, 8-10 weeks old), male Sprague Dawley rats (n = 3 per group, 8-10 weeks old) and male Beagle dogs (n = 3 per group, 12-14weeks old). Intravenous PK studies were done by administering 1 mg/kg EGT710 in a solution formulation containing 10% N-methyl-2-pyrrolidone (NMP) / 90% PBS with 4% BSA at 5 mL/kg dose volume in mice, 10% NMP / 90% PEG200 at 0.5 mL/kg dose volume in rats, and 0.2 mL/kg dose volume in dogs. The blood samples were collected between 0.083 h and 24 h post dosing in mouse and for up to 72 h post dosing in rats and dogs. Oral PK studies were done by administering 30 mg/kg EGT710 in a suspension formulation containing 0.5% methyl cellulose/0.1% Tween 80 in water at 10 mL/kg dose volume in mice and 10 mg/kg EGT710 in the same formulation at 2 mL/kg dose volume in dogs, and 10, or 100 mg/kg EGT710 in a formulation containing 0.5% methyl cellulose/0.5% Tween 80 in water at 5 mL/kg dose volume in rats. The blood samples were collected between 0.25 h and 24 h post dose in mice and between 0.25 h and 72 h post dose in rats and dogs. To support PK/PD study, uninfected Balb/c female mice (n = 3 per group, 8–10 weeks old) received a single oral dose of EGT710 at 1 mg/kg and 10 mg/kg to characterize the compound PK. EGT710 was administered orally using suspension formulation containing 0.5% methyl cellulose/0.5% Tween 80 in water at 10 mL/kg dose volume and blood samples were collected at various time points between 0 and 12 h post-dose. Concentration of EGT710 in whole blood was measured by LC-MS/MS as described in Supplementary Note. Pharmacokinetic parameters were determined by non-compartmental analysis using Phoenix WinNonLin v.8.3 (Certara).

### Prediction of human efficacious dose

Human clearance and volume of distribution for EGT710 were predicted using an average of relevant allometric scaling methods from mouse, rat, and dog PK data combined with the Wajima approach. To predict human PK following oral absorption of EGT710, predicted human disposition parameters were combined with physicochemical, solubility, and permeability data to build an advanced compartmental and transit (ACAT) physiologically based PK (PBPK) model in GastroPlus^TM^. After model validation for prediction of PK in dog following oral administration, this model was used to predict human bioavailability and absorption kinetics (see Supplementary Note).

To identify the EGT710 exposure target for efficacy, a previously developed viral kinetics model^15^ was re-calibrated for the delta variant and viral load reduction was validated by comparing predicted viral load reductions to clinical observations for other Mpro inhibitors. EGT710 doses that predicted comparable viral load reduction to treatment with nirmatrelvir + ritonavir were identified and the associated predicted Cmin was compared to EC_90_ to identify the required threshold for efficacious dose levels (see Supplementary Note).

### Toxicology studies

The rat and dog toxicology studies were conducted at designated test sites in compliance with principles of Good Laboratory Practice Standards. All procedures involving animals were reviewed and approved by the institutional animal care and use committees. Wistar Hannover rats (9 to 10 weeks of age, n = 10 per sex per group) were administered vehicle alone (0.5% methyl cellulose and 0.5% SLS in 50 mM acetate buffer pH 4.2) or a crystalline suspension of EGT710 at 10, 25, 75, or 250 mg/kg per day for 14 days by oral gavage. Beagle dogs (9 to 10 months of age, 7.4 to 9.6 kg for males and 6.1 to 8.5 kg for females, n = 3 per sex per group) were administered the vehicle alone (2% Kollidon VA64 and 0.1% SLS) or a crystalline nanosuspension of EGT710 at 30, 100, and 300/200 mg/kg per day for 14 days by oral gavage. Clinical observations, body weight, and food consumption determinations were performed on all animals in rat and dog groups. Clinical laboratory evaluations (hematology, coagulation, and clinical biochemistry) were performed at scheduled necropsy on day 15. In addition, jacket telemetry evaluations (pretest and study day 13) were performed on dogs. Gross pathology examinations were performed on all groups. Microscopic examinations were conducted on a standard list of vital organs and tissues. Details for safety pharmacology studies and developmental and reproductive toxicology studies are described in the Supplementary Note.

## Supplementary information

Supplementary Information

## Data Availability

All data associated with this study are present in the paper or the Supplementary Information. Co-structure of EGT710 with SARS-CoV-2 Mpro was deposited under PDB ID 9OIX; co-structure with HCoV-OC43 Mpro was deposited under PDB ID 9Y8X; co-structure with MERS-CoV Mpro was deposited under PDB ID 9Y8W.
