# Supplementary material for: Preclinical characterization of EGT710, an oral non-peptidomimetic reversible covalent SARS-CoV-2 main protease inhibitor
Source: NPJ Drug Discov. 2025 Nov 28;2:28. doi: 10.1038/s44386-025-00030-5 (PMC13267108; doi:10.1038/s44386-025-00030-5)
Supplement: Supplementary file 1 — Supplementary Information [file 44386_2025_30_MOESM1_ESM.pdf]

List of Supplementary Information

Supplementary Note

Supplementary figures

1. Figure S1. Structure of EGT710 in complex HCoV-OC43 Mpro and MERS-CoV Mpro.
2. Figure S2. In vitro biotransformation pathways of EGT710 in hepatocytes of mouse, rat, rabbit, dog and human
3. Figure S3. The one-state immune SARS-CoV-2 model recapitulates data digitized from [14], with subjects infected with the WT variant.
4. Figure S4. GastroPlus™-simulated vs observed dog EGT710 PK profiles for 1 mg/kg i.v. (left) and 30 mg/kg p.o. (as a nanosuspension)
5. Figure S5. The one-state immune SARS-CoV-2 model recapitulates viral load data from the placebo arm of the EMPATHY study, with patients predominantly infected with the delta variant.
6. Figure S6. The population predictions of viral load kinetics by the WT variant and delta variant models broadly overlap.
7. Figure S7. The SARS-CoV-2 delta variant viral kinetics model can reproduce the reported viral loads following treatment with placebo, nirmatrelvir+ritonavir, or ensitrelvir.

Supplementary tables

1. Table S1. In vitro activity of EGT710 against various coronavirus Mpro enzymes
2. Table S2. Additional mutations found in passaged viruses
3. Table S3. EGT710 as inhibitor or inducer of enzymes or transporters
4. Table S4. AHD table. Summary of predicted dose regimen across various conditions to achieve various EC_{90} targets
5. Table S5. Activity of EGT710 against human proteases
6. Table S6. Activity of EGT710 against a panel of ion-channels, transporters, enzymes, GPCRs etc. Target species include: human (h), rat (r), porcine (p). Abbreviations: GPCR, G-Protein Coupled Receptor; NHR, Nuclear Hormone Receptor, * % stimulation 10µM.
7. Table S7. Agilent RapidFire method parameters
8. Table S8. RapidFire-mass spectrometry assay reaction conditions
9. Table S9. Predicted human total clearance (CL) according to different allometric scaling methods

10. Table S10. Predicted human volume of distribution (Vss) according to different allometric scaling methods
11. Table S11. Parameters for 2-compartment PK model fit to the Wajima-predicted human i.v. systemic concentration-time profile for 1 mg/kg EGT710
12. Table S12. Input parameters for GastroPlus™ human PBPK model for EGT710
13. Table S13. Summary of model parameters for the WT SARS-CoV-2 variant
14. Table S14. Summary of model parameters for the SARS-CoV-2 delta variant

References (39-60)

Supplementary Note

Protein expression and purification

Mpro proteins from various coronaviruses were produced as reported in [39].

SPR

EGT710 was tested against SARS-CoV-2-His Mpro in covalent SPR mode using the Biacore 8K system (Cytiva). Prior to capturing SARS-CoV-2-His Mpro, biotinylated tris-NTA, at 1 µM in running buffer, was immobilized to a density of 300 response units (RUs) onto a Biacore S series SA chip that was preconditioned with 4X 60-second injections of 40 mM NaOH/1 M NaCl. Tris-NTA was activated by flowing 0.5 mM NiCl2 over the tris-NTA chip surface for 100 seconds at a flow rate of 10 µL/minute. Excess and weakly bound Ni(II) was removed by injecting 3 mM EDTA for 60 seconds at a flow rate of 10 µL/minute. Assay was conducted using SARS-CoV-2-His Mpro. The SARS-CoV-2-His Mpro protein was captured onto every channel to a 2500 RUs density by flowing 7 µM of protein for 50 seconds at a flow rate of 10 µL/minute. Capturing level coefficient of variation (CV) between all channels was less than 1%. Ten startup cycles and three blank cycles for double subtraction were ran prior to a dose response titration of NVP-EGT710 in parallel kinetics mode. After each run, tris-NTA chip surface regeneration was performed by making four 60-seconds injections in the following sequence at a flow rate of 10 µL/minute: 350 mM EDTA, 6 M urea/500 mM imidazole, 350 mM EDTA, 6 M urea/500 mM imidazole.

EGT710 was assayed in an 8-point, 3-fold serial dilutions with a top concentration of 100 nM. Testing was conducted in parallel kinetics mode (1 compound concentration/needle and 8 needle injections run simultaneously). Startup cycles, blank injections, and compound samples were injected for 280 seconds at a flow rate of 40 µL/minute with a dissociation time of 600 seconds. Data analysis was done using Biacore™ Insight Evaluation Software version 3.0.12.15655 (Cytiva). Binding kinetics was fitted to the 2-state reaction binding model.

RFMS

Compound mediated inhibition for the coronavirus Mpro enzyme panel was determined in a RapidFire-mass spectrometry (RFMS) assay using an Agilent RapidFire 365 autosampler (Agilent, Santa Clara, CA; method parameters listed in **Table S7**) coupled to Sciex 6500 triplequad (QQQ) mass spectrometer (Sciex, Framingham, MA). Mpro samples were diluted in assay buffer (50 mM HEPES, pH 7.3, 150 mM NaCl, 1 mM EDTA, 0.01% Pluronic® F-127) to 10 nM (2X final concentration). Five µL of Mpro enzyme was added into the assay ready plates dry-spotted with NVP-EGT710 (using 10 point, 3-fold serial dilutions with a top concentration of 50 µM dissolved in DMSO) via an Echo liquid handler (Labcyte, San Jose, CA) for 15 minutes prior to addition of substrate. To initiate the reaction, 2X substrate (10 µM) was added to the compound plate and incubated at room temperature between 2-3 hours depending on the Mpro enzyme tested (incubation times are listed in **Table S8**). Conditions were kept similar for each Mpro enzyme reaction with enzyme, substrate concentrations and reaction times adjusted to produce 10% substrate turnover in the absence of compound. After incubation, the reaction was quenched with 2% acetic acid solution and then submitted to the RFMS.

Samples on the RapidFire were loaded onto a C18 SPE cartridge (Agilent) in H_2O with 0.1% formic acid at a flow rate of 1.5 mL/minute. Samples were then eluted with 75:20:5 ACN:H_2O:IPA with 0.1% formic acid at a flow rate of 1.0 mL/minute.

Multiple reaction monitoring (MRM) transitions corresponding to the substrate and the related products, along with $^{13}C_3$ ^{15}N AVLQ (for signal normalization) were monitored, and peaks integrated using Sciex Multiquant (Sciex, Framingham, MA). Corresponding peptides (Vivitide, Gardner, MA) monitored for each Mpro enzyme are listed in **Table S8**.

Cells

VeroE6 cells were obtained from American Type Culture Collection (CRL-1586) and subsequently sorted and sub-cloned for high expression of ACE2, VeroE6-$ACE2_{high}$. Cells were maintained in DMEM supplemented with 10% FBS.

MRC5 cells, obtained from ATCC, were maintained in culture media (Minimum Essential Medium (MEM) supplemented with 10% FBS, 1% L-glutamine, 1% NEAA and 1% penicillin-streptomycin (Hyclone).

Huh7 cells were acquired from Wuxi AppTec and were maintained in culture media (DMEM supplemented with 10% FBS, 1% L-glutamine, 1% NEAA, and 1% penicillin-streptomycin).

Vero81 cells were obtained from ATCC (cat# CCL-81) and maintained in Dulbecco's Modified Eagle Medium, DMEM supplemented with 10% FBS.

Differentiated normal human bronchial epithelial (dNHBE) cells, also referred to as human airway epithelial (HAE) cells, were obtained from MatTek Life Sciences, Ashland, MA (MatTek Air-100). Maintenance media (MatTek Air-100-MM) on the basal side of cells was changed upon receipt of cells and subsequently once every two days. Cells were maintained at 37°C in 5% CO2 for 8-10 days before infection.

VeroE6-TMPRSS2 cells (SEKISUI XenoTech, LLC) were maintained in DMEM supplemented with 10% FBS, 1% Penicilin-Streptomycin and 0.5 mg/ml Geneticin. Human A549-hACE2 cells were generated at the University of Texas Medical Branch (UTMB) by transfecting A549 cells with human ACE2 (hACE2) and selecting a clonal stable cell line [40]. Cells were maintained in cell culture media DMEM supplemented with 10% FBS, 1% HEPES, 1% penicillin-streptomycin (10,000 U/mL).

Viruses

SARS-CoV-2 2019-nCoV/USA-WA1/2020 strain was obtained from the World Reference Center for Emerging Viruses and Arboviruses (WRCEVA) at the University of Texas Medical Branch.

HCoV-229E and HCoV-OC43 were acquired from ATCC.
MERS-CoV (Human Beta-Coronavirus Lineage C –Novel/2012) was obtained from Erasmus Medical Center.

SARS-CoV Toronto-2 strain was obtained from WRCEVA at the University of Texas of Medical Branch at Galveston.

SARS-CoV-2-Fluc was generated by engineering a firefly luciferase gene to replace ORF7 of the viral genome of the SARS-CoV-2 2019-nCoV/USA-WA1/2020 strain. The virus was generated as outlined by[40], except that firefly luciferase was used instead of nanoluciferase.

SARS-CoV-2 variants were generated using an infectious cDNA clone derived from clinical strain SARS-CoV-2 2019-nCoV/USA-WA1/2020 [41] as a template, and the recombinant viruses were cloned by using a PCR-based mutagenesis protocol as reported previously [26,42]. The genome

sequences of the virus stocks were confirmed to have no undesired mutations by Sanger sequencing as previously described [43]. All viruses were amplified on VeroE6-TMPRSS2 and titers were determined by plaque assay on VeroE6-TMPRSS2 cells. Accession numbers for the viruses are as follows B.1.1.7/alpha (EPI_ISL_999340), B.1.351/beta (EPI_ISL_678570), B.1.617.2/delta (EPI_ISL_2100646), and BA.1/omicron (EPI_ISL_2100646).
Recombinant viruses containing nsp5 mutations were engineered into the backbone of Δ3678 mNG SARS-CoV-2 according to a previous established protocol [9]. Briefly, nucleotide substitutions were introduced through a standard mutagenesis approach into a subclone pcc1-CoV-2-F123-TRS2 containing the nsp5 gene of the SARS-CoV-2 wildtype infectious cDNA clone of USA-/WA1-/2020. Overlapping PCR was performed to introduce nsp5 mutations (T21I, L50F, E166A and A191V) in plasmid pcc1-CoV-2-F123-TRS2. The full-length constructs were assembled by in vitro ligation of three contiguous cDNA fragments from plasmids pcc1-CoV-2-F123-TRS2, puc57-F4 and pcc1-F567-Δ3678 mNG following the previously described protocol [26,41]. In vitro transcription was then performed to synthesize full-length viral genomic RNA. For recovering mutant viruses, the RNA transcripts were electroporated in VeroE6-TMPRSS2 cells and harvested 2-5 days post electroporation. RNA was extracted and sequenced to confirm introduced mutations. The SARS-CoV-2 CMA4 strain is a clone-derived virus from CMA3p20 [24].

SARS-CoV-2 CPE assay in VeroE6 cells

VeroE6-ACE2high cells were harvested in assay media (DMEM supplemented with 2% FBS, 1% penicillin/streptomycin and 1% HEPES. Cells were batch inoculated with SARS-CoV-2 at a multiplicity of infection (MOI) equivalent to 0.002, which result in 5% cell viability at 72-hours post-infection. $4x10^3$ VeroE6 cells/well in 384-well plates were added to each well containing 90 nL of 10-point, 3-fold serially diluted compound (final DMSO concentration 0.3%). Dose responses were assessed in duplicate. Plates were incubated at 37°C, in 5% CO2 and 90% humidity for 72 h. Following the incubation period, 30 µL of Cell Titer-Glo® (Promega) was added to each well. Luminescence was read using a BMG CLARIOstar plate reader following incubation at room temperature for 10 min to measure cell viability. Cytotoxicity was assessed in parallel in uninfected cells using the same compound dilution, number of cells, and incubation time. To measure cell viability, 30 µL Cell Titer-Glo (Promega) was added to each well, and luminescence was read using a BMG PHERAstar plate reader following incubation at room temperature for 10 min. Cytotoxicity was assessed in singlicate. Compound concentration was plotted against luciferase and four parameter linear regression was performed using Helios software (Ref) to generate EC50 and CC50 values.

SARS-CoV-2 Fluc dNHBE assay

On the day of the assay, the apical side of the cells was washed for 30 min with Dulbecco's phosphate buffer saline (DPBS). Maintenance media on the basal side was replaced with 1 mL of assay media containing serial dilutions of EGT710. Each dilution was assayed in sextuplicate. 200 µL of diluted virus (180 µL DPBS + 20 µL virus stock) was added to the apical side of the cells and plates were incubated for 2 h at 37°C in 5% CO_2. Following the incubation, the inoculum was removed, and the apical side of the cells was rinsed with DPBS. Cells were incubated at 37°C and 5% humidity and washed once daily with DPBS on the apical side for 30 min. Three days post-infection, cells were briefly rinsed once with DPBS, and were lysed in 300 µL of 1X lysis buffer (Promega; cat# E1531) for 20 min. The luciferase assay was performed on the cell lysate as follows: 30 µL of lysate was transferred into two separate wells of a 96-well plate for technical

replicates, and 60 μL of luciferase substrate was added to each well and incubated at room temperature for 2-5 min. Luciferase was measured using the Synergy Neo2 (BioTek). Cytotoxicity was assessed in parallel in uninfected cells using six wells at the highest compound concentration tested in the antiviral assay (1 μM), the same compound dilution, cell number, and incubation time. To measure cell viability, 30 μL of lysate was transferred into two separate wells of a 96-well plate for technical replicates, and 60 μL of CellTiter-Glo substrate (Promega) was added to each well and luminescence was measured using the Synergy Neo2 (BioTek). Compound concentration was plotted against luminescence and four parameter linear regression was performed using Helios software (Ref) to generate EC_{50} values.

HCoV-229E CPE assay in MRC5 cells

MRC5 cells were seeded in 96-well plates, in 100 μL per well of assay medium (MEM supplemented with 5% FBS, 1% L-glutamine, 1% NEAA and 1% penicillin-streptomycin), at a density of $2x10^4$ cells/well. Plates were incubated overnight at 37°C in 5% CO2. EGT710 was diluted to prepare an 8-point, 3-fold dilution series in DMSO in assay medium and added to the cells (50 μL per well). Each compound concentration was assayed in duplicate. 50 μL of assay medium-diluted virus was added to each well for a final MOI of approximately 0.007. The final volume of the cell culture was 200 μL per well. The final concentrations of DMSO in the assay was 0.5%. Cells were incubated at 35°C in 5% CO2 for an additional 3 days at which point the virus infection observed in the virus control wells (untreated infected cells) displayed significant CPE. The CPE was measured by CellTiter Glo® (Promega) according to the manufacturer's instructions. Cytotoxicity was assessed in parallel in uninfected cells using the same compound dilution, number of cells, and incubation time. Cell viability was measured with CellTiter Glo® following the manufacturer's manual. Compound concentration was plotted against luminescence and four parameter linear regression was performed using Helios software (Ref) to generate EC50 and CC50 values.

HCoV-OC43 CPE assay in Huh7 cells

Huh7 cells were seeded in 96-well plates, in 100 μL per well of assay medium, at a density of $8x10^3$ cells/well. Plates were incubated overnight at 37°C in 5% CO2. EGT710 was diluted with assay medium and added into the cells (50 μL per well). Each compound concentration was assayed in duplicate. 50 μL of assay medium-diluted virus was added to each well for a final MOI of approximately 0.00875. The final volume of the cell culture was 200 μL per well. The final concentrations of DMSO in the assay was 0.5%. The resulting cell culture were incubated at 33°C in 5% CO2 for an additional 7 days at which point the virus infection observed in the virus control wells (untreated infected cells) displayed significant CPE. The CPE was measured by CellTiter Glo® (Promega; cat# G9243) according to the manufacturer's instructions. Cytotoxicity was assessed in parallel in uninfected cells using the same compound dilution, number of cells, and incubation time. Cell viability was measured with CellTiter Glo® following the manufacturer's manual. Compound concentration was plotted against luminescence and four parameter linear regression was performed using Helios software (Ref) to generate EC50 and CC50 values.

MERS-CoV CPE assay in Vero81 cells

MERS-CoV assay was performed the same as SARS-CoV-2 assay except using Vero81 cells, MEM with 2% FBS and 1% penicllin-streptomycin (Gibco) as the assay media, infection with MERS-CoV at an MOI equivalent to 0.01, and an assay duration of 96 hours.

SARS-CoV-2 Fluc A549-ACE2 assay

A549-hACE2 cells were collected and resuspended in assay media (DMEM media supplemented with 2% FBS, 2% GlutaMAX™ Supplement (Gibco), 1% HEPES and 1% penicillin-streptomycin (10,000 U/mL). A total of 1.2×10^4 cells suspended in 50 µL assay media were seeded in each well of a 96-well white opaque plate (Corning; cat# 3916), and plates were incubated overnight at 37°C in 5% CO_2. On the day of the assay, SARS-CoV-2-Fluc was diluted to a concentration of 2.5×10^4 PFU/mL using assay media, and 100 µL of diluted virus was added to 1 µL of serially diluted compound. 50 µL of the compound-virus mixture from a single well were added to A549-hACE2 cells, resulting in an MOI of approximately 0.1. After 1 h infection, the inoculum was replaced by 100 µL of fresh assay media containing 0.5 µL of 9-point 3-fold compound serially diluted in DMSO. Dose responses were assessed in duplicate. The plates were incubated at 37°C. At 48 h post-infection, 100 µL of Bio-Glo™ Luciferase Assay System substrates (Promega; cat# G7940) was added to each well. Luciferase signals were measured using a Synergy™ Neo2 Multi-Mode microplate reader (BioTek, San Diego, CA). Assays were conducted in duplicate. Cytotoxicity was assessed in parallel in uninfected cells using the same compound dilution, number of cells, and incubation time. To measure cell viability, 50 µL Cell Titer-Glo (Promega) was added to each well, and luminescence was read using a Synergy™ Neo2 Multi-Mode microplate reader (BioTek). Compound concentration was plotted against luminescence and four parameter linear regression was performed using Helios software [44] to generate EC_{50} and CC_{50} values.

SARS-CoV-2 variants in A549-ACE2 assay

A549-hACE2 cells were collected and resuspended in assay media (DMEM media supplemented with 2% FBS, 1% HEPES and 1% penicillin-streptomycin (10,000 U/mL)). A total of 1.2×10^4 cells suspended in 50 µL assay media were seeded in each well of a 96-well white opaque flat-bottom plate, and plates were incubated overnight at 37°C in 5% CO_2. On the day of the assay, SARS-CoV-2 variants were diluted to a concentration of 2.5×10^4 PFU/mL using assay media, and 100 µL of diluted virus was added to 1 µL of compound serially diluted in DMSO (7-point, 3-fold dilution). 50 µL of the compound-virus mixture from single wells were added to A549-hACE2 cells, resulting in an MOI of approximately 0.1. After 1 h infection, the inoculum was replaced by 100 µL of fresh medium assay media containing 0.5 µL of serially diluted compound. The plates were incubated at 37°C for 48 h. At 48 h post-infection, 100 µL of supernatants were collected from each well and mixed with 500 µL of TRIzol™ LS. RNAs were extracted by the Direct-zol™ RNA Miniprep Plus kit (Zymo Research, Irvine, CA; cat# R2072) and eluted in 50 µL RNase-free water. qRT-PCR was performed as described above to quantify the viral RNA copies. The relative RNA levels were calculated by normalizing the cycle threshold (Ct) values to the infected DMSO-treated control (DMSO control was treated as 100%). Four-parameter non-linear regression was performed using the "log(inhibitor) vs. response – Variable slope (four parameters)" option in GraphPad Prism version 9.3.1 (GraphPad Software, San Diego, CA). The EC_{90} value was calculated in Microsoft Excel using the EC_{50} value, the Hill slope (h), and the equation $EC_{90} = EC50 \text{ x } (90/100\text{-}90)^{(1/h)}$.

SARS-CoV-1 CPE assay in VeroE6 cells

VeroE6-ACE2$_{high}$ cells were harvested in assay media (DMEM supplemented with 2% FBS, 1% penicillin/streptomycin and 1% HEPES. Cells were batch inoculated with SARS-CoV at a multiplicity of infection (MOI) equivalent to 0.01. $4x10^3$ VeroE6 cells/well were added to each well containing 90 nL of 10-point, 3-fold serially diluted compound (final DMSO concentration 0.3%). Dose responses were assessed in duplicate. Plates were incubated at 37°C, in 5% CO_2 and 90% humidity for 72 h. Following the incubation period, 30 µL of Cell Titer-Glo® (Promega) was added to each well. Luminescence was read using a BMG CLARIOstar plate reader following incubation at room temperature for 10 min to measure cell viability. Cytotoxicity was assessed in parallel in uninfected cells using the same compound dilution, number of cells, and incubation time. To measure cell viability, 30 µL Cell Titer-Glo (Promega) was added to each well, and luminescence was read using a BMG PHERAstar plate reader following incubation at room temperature for 10 min. Cytotoxicity was assessed in singlicate. Compound concentration was plotted against luciferase and four parameter linear regression was performed using Helios software to generate EC_{50} and CC_{50} values.

Next generation sequencing analysis

The de novo assembly program ABySS version 2.3.1 [45] was used to assemble the reads into contigs, using the default paired-end setting, using several different subsets of reads, from 25,000 to 2 million read-pairs, and kmer values from 19 to 40. Related contigs were grouped and the longest example from each group was selected using cd-hit software version 4.8.1 [46], using the following parameters: -G 0 -aL 0.01 -aS 0.4 -c 0.95 -n 8 -r 1. Reads were mapped back to the viral contigs using bowtie2 with the –local parameter version 2.3.4.1 [47] and visualized with the Integrative Genomics Viewer [48] to verify that the assembled contigs were correct. Assembly errors were corrected and contig ends were extended using the mapped reads. Single nucleotide variants (SNVs) and short insertion/deletions were called using the open-source software LoFreq V2 [49]with a variant frequency cutoff of 0.5%. For analysis of longer insertion/deletions, reads were mapped to the reference sequence using BBMap version 38.90 [50] with the maxindel parameter set to 1000. Insertion/deletion calls were made with LoFreq V2 version 2.1.3.1 [49] .

GISAID sequence prevalence

A total of 17,097,741 SARS-CoV-2 sequences were downloaded from the Global Initiative on Sharing Avian Influenza Data (GISAID, gisaid.org) database on January 6th, 2025. Using custom python code, isolates were filtered for quality using the following criteria: (1) at least 95% of the nsp5 sequence was found in the isolate following reference alignment (2) No more than 10 ambiguous amino acids were present in the nsp5 sequence, as assessed by the corresponding codon containing an "N" base (3) the FASTA header followed an expected regular expression format (e.g., expected number of columns, correct data in each field). After filtering, a total of 15,593,310 sequences were retained. Sequences were aligned, and across all isolates, the number and fraction of amino acids different from the reference were calculated at each reference position.

In vivo pharmacokinetic sample analysis

Whole blood samples were thawed over wet ice and an aliquot of each blood sample was transferred into a new 96-well plate. Proteins were precipitated from these samples by adding acetonitrile extraction solution containing internal standard. The plate was covered, swirled gently for 30 seconds, and centrifuged for 10 min at 4000 rpm at 10°C. 50 µL of supernatant from each sample was transferred into a fresh high performance liquid chromatography (HPLC) 96-well injection plate. 50 µL of deionized water was added to all samples in the injection plate and gently

swirled. The samples were then analyzed for compound concentration. Analyte quantitation was performed by HPLC coupled with tandem mass spectrometry (LC-MS/MS). For mice PK, liquid chromatography was performed using an Agilent 1260, CTC PAL autosampler system, with ACE C18, 50x2.1mm, 1.7µM column at an oven temperature of 45ºC, coupled with a API5500 triple quadrupole mass spectrometer. The mobile phases used were (A) 0.1%formic acid in water and (B) 0.1% formic acid in acetonitrile, using a gradient, with flow rate of 0.8 mL/min, and run time of 2.5 min. For rat and dog PK, liquid chromatography was performed using an Sciex Exion AD autosampler system, with Acquity BEH C18, 50x2.1mm, 1.7µM column at an oven temperature of 50ºC, coupled with a API6500+ triple quadrupole mass spectrometer. The mobile phases used were (A) 0.1%formic acid in water and (B) 0.1% formic acid in acetonitrile, using a gradient, with flow rate of 0.8 mL/min, and run time of 2 min. For samples from mouse PK/PD study, liquid chromatography was performed using a Waters ultra-performance liquid chromatography (UPLC) system, with ACQUITY UPLC HSS C18 SB column at an oven temperature of 50ºC, coupled with a API6500 triple quadrupole mass spectrometer. The mobile phases used were (A) water-acetonitrile-formic acid (94.9:5:0.1, v/v/v %) and (B) water-acetonitrile-formic acid (5:94.9:0.1, v/v/v %), using a gradient, with flow rate of 0.6 mL/min, and run time of 2.4 min. Multiple-reaction monitoring (MRM) was combined with optimized mass spectrometry parameters to maximize detection specificity and sensitivity. The most intense MRM transitions (384.0 / 357.4 for EGT710 and 255.1 / 153.0 for the internal standard Chyrsin) were used for quantification. Compound detection on the mass spectrometer was performed in electrospray positive ionization mode. Injection volume was 4-10 µL. For EGT710, the lower limit of quantification was 0.1 - 1 ng/mL A calibration curve was freshly prepared and analyzed with every set of study samples. Intra-day variability was established with triplicate quality control samples at three concentration levels. The results were accepted if relative standard deviation was within 30%.

Prediction of human pharmacokinetics

EGT710 disposition in human disposition was predicted using allometric scaling of CL (**Table S9**; [51]) and V_{ss} (**Table S10**;[52]) and the Wajima approach [53], assuming bodyweights of 0.025, 0.25, 10, and 70 kg for mouse, rat, dog, and human, respectively. Fitting a 2-compartment model to the Wajima-predicted human i.v. concentration-time profile yielded the disposition parameters listed in **Table S11**.

To predict the oral absorption of EGT710 in human, an advanced compartmental absorption and transit (ACAT) model was built in GastroPlus™ (v.9.8). Briefly, the use of the ACAT model along with a 2-compartment disposition PK model was validated using dog p.o. PK data. EGT710 physico-chemical properties, in vitro solubility data, and formulation information (**Table S12**) were used to parametrize a model in GastroPlus™. The ability of this model to recapitulate EGT710 PK following p.o. administration of 30 mg/kg EGT710 in nanogranule formulation in dog was assessed visually (**Fig. S4**). The GastroPlus™ model predicted a liver first-pass effect (FPE) of 19% for the dog (thus F~80%). Using the thus-qualified physico-chemical properties of EGT710 and the predicted human disposition parameters, a human - model was constructed. When using human physiology, GastroPlus™ predicted a liver FPE of ~10%. However, considering that EGT710 is predicted to be a CYP3A4 inducer in human, an additional 15% FPE was conservatively assumed (**Table S12**) in the final translational PBPK model.

Overall, given that:

1) the measured in vitro solubility of EGT710 in aqueous media is low with a reference solubility of 0.010 mg/mL at pH 6.8, as a base with measured pKa~4.2,
2) the classification of EGT710 as a DCS IIb compound,
3) the decrease of the fraction of drug absorbed with increasing doses predicted by the human EGT710 ACAT model in GastroPlus™,

EGT710 absorption is likely to be limited by its solubility. Therefore, all predictions of EGT710 concentration-time profiles and exposures in human were performed using the GastroPlus model to account for the predicted non-linearities with increasing oral doses.

Prediction of drug-drug interaction liabilities

According to the net effect model and the static DDI assessment (**Table S3**), in vitro DDI flags for CYP3A4, CYP2C8 and CYP1A2 induction as well as P-gp, OCTs and MATEs inhibition were further assessed by dynamic PBPK modeling. Based on the disposition parameters of the final translational PBPK model, a minimal human PBPK model was developed in Simcyp v21.1 (Certara Inc., Princeton, NJ). It was used to predict the DDI potential of EGT710 as inhibitor and inducer of enzymes and transporters at 600 mg QD dose in healthy volunteers. For that purpose, the PBPK model was informed with the in vitro interaction properties of EGT710 as listed in **Table S3**. PBPK DDI trial simulations were performed with a 10 trial x 10 subjects (n=100) design using the Simcyp Healthy volunteer population with age range of 20-50 years and assuming equal proportion of male and female subjects. In PBPK simulations, EGT710 600 mg was orally administered for 7 days once daily, while a single oral dose of 4 mg midazolam (CYP3A4 substrate), 150 mg caffeine (CYP1A2 substrate), 0.25 mg repaglinide (CYP2C8/ CYP3A4 substrate), 0.5 mg digoxin (P-gp substrate) or 500 mg metformin (OCTs/ MATEs substrate) were given on day 5. The PBPK model indicated a moderate effect for CYP3A4 and CYP2C8 induction predicting a 65% and 32% decrease in midazolam (AUC ratio of 0.35) and repaglinide (AUC ratio of 0.68) exposure, respectively. On the other hand, a weak inhibition effect on P-gp was predicted increasing digoxin exposure and C_{max} by 37% and 87%, respectively. No clinically relevant effects (i.e., 0.8 <AUC or C_{max} ratio < 1.25) were predicted for caffeine or metformin. Nevertheless, due to the complexity of elimination pathways of metformin, the potential of MATEs or OCTs inhibition by EGT710 in vivo cannot be excluded.

SARS-CoV-2 viral kinetics model

WT variant model. The one-state immune viral kinetics SARS-CoV-2 model structure as described in [15] was used. Briefly, the final model structure describes viral particles, *V*, that infect uninfected cells, *f* (i.e., the fraction of cells still uninfected, where $f = U/U_0$), at rate *beta*. Once a cell is infected, *I*, it produces virus particles at rate $p \cdot I$; infected cells and viral particles are cleared at rates $delta \cdot I$ and $c \cdot V$ respectively. Infected cells further give rise to an immune response, *X*, which both inhibits production of viral particles (with factor $epsilon \cdot X$) and promotes clearance of infected cells with rate $eta \cdot X \cdot I$. The model equations are:

$$\frac{df}{dt} = -beta \cdot f \cdot V \tag{1}$$

$$\frac{dI}{dt} = beta \cdot f \cdot V - delta \cdot (1 + eta \cdot X) \cdot I \tag{2}$$

$$\frac{dV}{dt} = \frac{p \cdot I}{1 + epsilon \cdot X} - c \cdot V \tag{3}$$

$$\frac{dX}{dt} = I \tag{4}$$

To parametrize the model, first, a WT variant base model was built using data from a challenge study in which individuals were infected with controlled levels of SARS-CoV-2 and monitored following exposure to measure viral load [14]. These data capture the early viral kinetics from infection time to peak viral load, which is often concurrent with symptom onset; these data important for the model-based understanding of the early phase of infection.

Observed viral load data [14] for individual viral load kinetics in nasopharyngeal swabs for the 12 infected patients who were not treated with additional therapeutic intervention were digitized (WebPlotDigitizer v4.4 [54]) and converted to virus concentrations, as described in [15]. Nasopharyngeal swabs had initial virus quantifiable on average 2.4 days after inoculation with peak viral load at 6.2 days data [14]. In general, the behavior is biphasic with an exponential increasing and decreasing phase (shown as linear in log space), with more data-points capturing the decreasing phase and larger associated variability.

Model estimation was performed in Monolix 2020R1 (Lixoft, Antony, France) with final model selection criteria being defined as 1) observable convergence of the Stochastic Approximation Expectation-Maximization algorithm, 2) an identifiable model (manifested as an estimable Fisher information matrix), and 3) minimized Akaike Information Criterion (AIC) value. Multiple model structures were explored with varying random effects and error models, with the final selected model composing of random effects on beta and eta and a constant error model.

Multiple steps were taken to identify and parameterize such a model. In Step 1, a base parameter set was determined by manually testing initial conditions to yield good predicted-versus-observed behavior and minimized Akaike Information Criterion (AIC) and Bayesian Information Criterion (BIC) values. At this step it was observed that the estimated model parameters were sensitive to initial values and the Fisher information matrix could not be approximated. Step 2 of parameter estimation was to initiate 1000 semi-random start sites to generate a family of parameter sets that could describe the individual data to a similar extent. Leveraging the parameter set optimized in Step 1, parameter sets were generated by sampling a log-normal distribution generated around each optimized parameter value, assuming independence. The log-normal distributions were set with mean *mu* = log10(parameter) and standard deviation *sigma* = 1, which equates to a standard deviation of one order of magnitude above and below the originally optimized value. At this stage, 60 out of 1000 model fits resulted in identifiable models. After identifying that the top model fits were at the boundary of the distribution of initial parameter values, step 3 was a refinement step wherein the best fit model from step 2 was selected and 1000x semi-random start sites as defined above were executed. From these 1000 model fits, 230 were identifiable and 41 were within 3 AIC units of the best fit model as defined by the model with the smallest absolute AIC value. Upon comparing the best fit model to the 41 models within 3 AIC units (assumed to be statistically indistinguishable), all parameters except for *epsilon* were over-approximated with the population model compared to the range estimated in the pool of models. As such, it is assumed that population variability is larger than the inter-model variability in parameters and the best fit model was used as a representative base viral kinetic model of the WT variant.

Model fits to the data from [14] are shown in **Fig. S3 (top)**. They support the consistency between the model structure selected and the reported patient data. Furthermore, the individual predictions versus observations shown in **Fig. S3 (bottom)** support the validity of the model fit as the values are distributed uniformly around the unity line. Additional qualitative checks from the model fit were the ability of the algorithm to estimate the Fisher information matrix and the estimation of all parameters, criteria achieved by this model. The parameter estimates (**Table S13**) show all parameter values being estimable with low residual squared error (R.S.E.) between <1-15% except *eta* which had a moderately high R.S.E. value. Nonetheless, these data support the development and selection of a final WT variant model suitable for describing the viral kinetics after infection with the WT variant of SARS-CoV-2.

Delta variant model. To expand the applicability of the model, a delta variant model was generated by fitting the model to viral load data from the placebo arm of the EMPATHY clinical trial (n = 94 subjects;[16]. As day 1 in the study represents the first day of treatment and there is a delay from infection to symptom onset to diagnosis via rapid antigen testing and then treatment, we aligned time t = 0 to be the day of positive diagnosis via rapid antigen testing to eliminate one source of time-variability among patients.

To estimate the delta variant model, model learnings and parameters from the WT variant model were used as prior information. Parameters *p* (rate constant for virus production by infected cells), *delta* (rate constant for clearance of infected cells), *c* (native clearance of virus), *epsilon* (effect of immune response on viral production rate), and *Omega_beta* were fixed to the estimated values from the WT variant model (**Table S13**). To estimate the remaining parameters, prior distributions for *beta* (rate constant of infection), *eta* (rate constant for viral clearance by immune response) and *Omega_eta* were set such that the typical value was the population estimate from the WT variant model and the standard deviation was 0.1X the typical value (equivalent to a coefficient of variation (CV) of 0.1). Finally, to account for the fact that the EMPATHY dataset designates day 1 as the first day of treatment (offset from infection time), an offset time parameter, *t_init*, was introduced to represent time from infection until dosing initiation. Then, *t_init* was set to 8 days, based on testing different time offset values and assessing model-data alignment by overlaying population simulations from the WT model variant with the placebo treated data from the EMPATHY study. This assumes that the time from infection to treatment is the same for delta vs WT variant. Based on this model, 100 semi-random start sites as defined above were initiated and all resulting model fits were identifiable with well-constrained parameter distributions.

The delta variant model fit the data well (**Fig. S5, top**), with the empirical 10th, 50th, and 90th percentiles (solid lines, bottom to top respectively) are within the model 90th percentile prediction intervals (shaded regions) at each data percentile region (except one outlier). Individual predictions versus observations are distributed around the unity line in (**Fig. S5, bottom**).

As with the WT variant model, population parameter variability was larger than parameter values across model fits within 3 AIC units of the best fit model. Furthermore, the WT model optimized to the Kingsley J *et al.* (2020) data and the delta variant model optimized the EMPATHY placebo data have population predictions that are largely overlapping with subtle shifts in mean predictions (**Fig. S6**). Due to the more immediate relevance of the delta variant as well as the larger population size (n = 94 for EMPATHY placebo data vs. n = 12 for challenge study), the delta variant model was used for all simulations with anti-replicative treatments. Viral loads and viral load kinetics of the delta and omicron variants of SARS-CoV-2 have been reported to be similar [55,56] and therefore

the delta variant model is expected to be valid for multiple variants. The final parameters for the delta variant model are listed in **Table S15**.

Anticipated human dose projection

Modeling the Mpro mechanism of action. To integrate the effects of an Mpro inhibitor, an anti-replicative therapeutic intervention was added to the viral kinetics model. The mechanism of action was assumed to be a direct effect on parameter *p*, described by a sigmoidal relationship with drug concentration in plasma:

$$\alpha = 1 - \frac{C^h}{C^h + EC50^h} \tag{5}$$

The free drug potency is assumed to be the same in vitro and in vivo, and thus the drug-specific potency, *EC50*, and Hill coefficient, *h*, are derived from in vitro concentration-response curves in cellular assays, accounting for protein binding in plasma and/or assay medium.

Benchmarking to nirmatrelvir+ritonavir. To generate model predictions for benchmarking against nirmatrelvir+ritonavir, multiple data sources were used:

1. Reported mean human PK data for PF-07321332 (nirmatrelvir) after treatment with 250 mg nirmatrelvir + 100 mg ritonavir [1] digitized (WebPlotDigitizer v4.4[54]) and fit to a 1-compartment PK model assuming linear absorption and elimination kinetics.
2. PK model simulations (**Fig. S7A**) were checked for agreement with mean model prediction for nirmatrelvir after treatment with 300 mg nirmatrelvir + 100 mg ritonavir, digitized (WebPlotDigitizer v4.4[54]) from ([13]), supporting the PK model assumptions.
3. In vitro potency reported for dNHBE cells [1] was used. The *EC50,u* (61.8 nM) was combined with plasma protein binding (with F_{up} = 31%) and molecular weight (499.5 g/mol) [1] , to convert *EC50,u* to $EC50_{total}$ for compatibility with total PK predictions yielding $EC50_{total}$ = 99.6 ng/mL. To derive *h*, Equation (6) below was used, with EC_{50} (99.6 ng/mL) and EC_{90} (292 ng/mL; [1]), to yield *h* = 2.04. Ritonavir was assumed to have no anti-viral activity and to have a constant effect on modulating nirmatrelvir PK; ritonavir PK and anti-viral effects were not explicitly modeled.

$$h = \frac{\log(9)}{\log\left(\frac{EC90}{EC50}\right)} \tag{6}$$

Nirmatrelvir simulations were based on the 300 mg nirmatrelvir + 100 mg ritonavir twice daily (BID) regimen used in the EPIC-HR trial. Model predictions were compared to reported mean viral load change from baseline as reported in the EUA application [13] (**Fig. S7B**).

Benchmarking to ensitrelvir. To generate model predictions for benchmarking against ensitrelvir, multiple data sources were used:

1. Human PK data from Japanese and Caucasian COVID19 patients in a Phase 2a trial presented in a publicly available presentation [57]were digitized (WebPlotDigitizer v4.4 [54]). PK data were modeled independently for Japanese and Caucasian participants by fitting to a 2-compartment PK model assuming linear absorption and clearance.
2. In vitro potency metrics EC_{50} and Hill coefficient *h* were determined by digitizing (WebPlotDigitizer v4.4;[54]) in vit*o* data in VeroE6/TMPRSS2 cells [2] and fitting a four-

parameter logistic regression model. Omicron variant values were used, to reflect the population treated in the reported Ph2b clinical results. To convert in vitro $EC50,u$ (0.37 µM) to $EC50_{total}$, human plasma protein binding was measured (96.7%), yielding $EC50_{total}$ = 4673 ng/mL, using the molecular weight of 531.9 g/mol. From the four-parameter logistic regression model, h = 2.34 was estimated.

Ensitrelvir simulations were based on the 375 mg loading dose + 125 mg QD for 5 days total (375/125) and 750 mg loading dose + 250 mg QD for 5 days total (750/250) dose regimen used in the Ph2b trial. Model predictions were compared to reported mean viral load change from baseline[58], later published in [59] (**Fig. S7D**).

EGT710 human efficacious dose prediction. To generate an EGT710-specific model, parameters governing the anti-replicative behavior of EGT710 were updated in the model. In vitro EC_{50} and EC_{90} were assessed in dNHBE cells (**Fig. 1E**). These values were corrected by fraction unbound in assay considering 2.82% bound in dNHBE cell medium (**Table 1**), and by human plasma protein binding (88.2%; **Table 1**) to obtain $EC50_{total}$ = 194 nM (74 ng/mL) and $EC90_{total}$ = 585 nM (224 ng/mL). Using these values and Equation 6 yielded h = 1.98.

Model simulations were performed with doses targeting $C_{trough,24h}$ (at t = 24 h post treatment initiation) values of 1X to 5X $EC90_{total}$ to establish the dose-response behavior of EGT710, assuming a 5-day regimen. PK profiles were obtained from the EGT710 PBPK model using fasted or fed (high fat high carbohydrate meal condition in GastroPlus™). Dose initiation was simulated on day 8 post-infection, consistent with alignment of day 1 of treatment in the delta variant EMPATHY placebo data [16] with day 8 post-infection from the WT variant data[14]. Simulations were performed with the delta variant viral kinetic model, with a virtual population of n=1000. Mean response metrics on total viral load (V_{out}) and viral load change from baseline were calculated and predictions were compared to those made for nirmatrelvir+ritonavir and ensitrelvir efficacious regimens.

Safety pharmacology studies

Cardiovascular (CV) function was assessed in instrumented male beagle dogs (n=4/group). A telemetry implant (Data Sciences International; PhysioTel Digital L11) was inserted via surgical procedure along with the catheters/leads for CV measurements at least 2 weeks before dosing. Telemetry data were collected after single oral doses of 30, 100 or 200 mg/kg EGT710 and transferred to a Ponemah (Ponemah Physiology Platform-Plus) analysis system.

Central nervous system (CNS) and respiratory function were evaluated in male Wistar Han rats after a single dose of 250 mg/kg. For CNS effects, a modified Irwin screen was employed (n=10 rats/group) and evaluated after 2, 6 and 24 hours post-dose. Respiratory evaluations were done in a cross-over study design (n= 5 rats/group) on Days 1 and 8. Tidal volume, respiration rate and minute volume were evaluated using head-out plethysmography continuously from dosing through 6 hours postdose, and again at 24 hours post-dose.

Developmental and reproductive toxicology studies

Developmental and reproductive toxicity studies were conducted in rats and rabbits using the following vehicle - 0.5% methyl cellulose and 0.5% sodium lauryl sulphate in 50 mM acetate buffer pH 4.2.

In fertility studies, Wistar Hannover rats (n = 20 per group) were administered vehicle alone or EGT710 at 30, 100, or 300 mg/kg per day. Male rats were treated 28 days prior to mating, during the 2-week pairing phase until termination while the female rats were treated 14 days prior to mating, during the 2-week pairing phase until gestation day (GD) 6. Clinical observations, body weight, and food consumption determinations were performed. Cesarean section was performed on GD13. Pregnancy status was determined, and the uterus were examined for the number of live and dead embryos, number of resorptions and any abnormalities. The number of corpora lutea was examined in both ovaries.

In a rat embryo fetal development study, pregnant rats (n=22 per group) were administered vehicle alone or EGT710 at 30, 100, or 300 mg/kg per day from GD 6 to GD 17 and cesarean sectioned on GD 21. In a rabbit embryo fetal development study, pregnant rabbits (n=20 per group) were administered vehicle alone or EGT710 at 25, 50 or 100 mg/kg per day from GD 7 to GD 20 and cesarean sectioned on GD 29. Clinical observations, body weight, and food consumption determinations were performed. Cesarean section observations included gravid uterine weights, ovarian corpora lutea counts, implantations, fetuses (live and dead), early and late resorptions. Fetuses were examined for any external, skeletal, and visceral abnormalities.

References

39. Barkan, D. T. *et al.* Identification of Potent, Broad-Spectrum Coronavirus Main Protease Inhibitors for Pandemic Preparedness. *J Med Chem* (2024) doi:10.1021/acs.jmedchem.4c01404.

40. Xie, X. *et al.* A nanoluciferase SARS-CoV-2 for rapid neutralization testing and screening of anti-infective drugs for COVID-19. *Nat Commun* **11**, 1–11 (2020).

41. Xie, X. *et al.* An Infectious cDNA Clone of SARS-CoV-2. *Cell Host Microbe* **27**, 841-848.e3 (2020).

42. Liu, Y. *et al.* Delta spike P681R mutation enhances SARS-CoV-2 fitness over Alpha variant. *Cell Rep* **39**, 110829 (2022).

43. Xie, X. *et al.* Engineering SARS-CoV-2 using a reverse genetic system. *Nat Protoc* **16**, 1761–1784 (2021).

44. Gubler, H. *et al.* Helios: History and Anatomy of a Successful In-House Enterprise High-Throughput Screening and Profiling Data Analysis System. *SLAS Discovery* **23**, 474–488 (2018).

45. Simpson, J. T. *et al.* ABySS: A parallel assembler for short read sequence data. *Genome Res* **19**, 1117–1123 (2009).

46. Li, W. & Godzik, A. Cd-hit: A fast program for clustering and comparing large sets of protein or nucleotide sequences. *Bioinformatics* **22**, 1658–1659 (2006).

47. Langmead, B. & Salzberg, S. L. Fast gapped-read alignment with Bowtie 2. *Nat Methods* **9**, 357–359 (2012).

48. Robinson, J. T. *et al. Integrative Genomics Viewer*. *Nature Publishing Group* http://maq.sourceforge.net/ (2011) doi:10.1038/nbt0111-24.

49. Wilm, A. *et al.* LoFreq: A sequence-quality aware, ultra-sensitive variant caller for uncovering cell-population heterogeneity from high-throughput sequencing datasets. *Nucleic Acids Res* **40**, 11189–11201 (2012).

50. Bushnell, B. BBMap Available from: <https://sourceforge.net/projects/bbmap> (Accessed 02 FEB 2021).

51. Ring, B. J. *et al.* PhRMA CPCDC initiative on predictive models of human pharmacokinetics, part 3: Comparative assessment of prediction methods of human clearance. *J Pharm Sci* **100**, 4090–4110 (2011).

52. Jones, R. Do *et al.* PhRMA CPCDC initiative on predictive models of human pharmacokinetics, part 2: Comparative assessment of prediction methods of human volume of distribution. *J Pharm Sci* **100**, 4074–4089 (2011).

53. Vuppugalla, R. *et al.* PhRMA CPCDC initiative on predictive models of human pharmacokinetics, part 4: Prediction of plasma concentration-time profiles in human from in vivo preclinical data by using the Wajima approach. *J Pharm Sci* **100**, 4111–4126 (2011).

54. Rohatgi, A. Webplotdigitizer: version 4.4. Available at: https://automeris.io/WebPlotDigitizer. (2020).

55. Hay, J. A. *et al.* Quantifying the impact of immune history and variant on SARS-CoV-2 viral kinetics and infection rebound: A retrospective cohort study. *Elife* **11**, (2022).

56. Puhach, O. *et al.* Infectious viral load in unvaccinated and vaccinated individuals infected with ancestral, Delta or Omicron SARS-CoV-2. *Nat Med* **28**, 1491–1500 (2022).

57. Yotsuyanagi, H. *et al.* Results from Ph1 and Ph2a studies of S-217622, a novel 3C-like protease inhibitor as once daily oral treatment for SARS-CoV-2 infection. *32nd European Congress of Clinical Microbiology and Infectious Diseases. Lisbon, Portugal* (2022).

58. Mukae, H. *et al.* Efficacy and safety of ensitrelvir in patients with mild-to-moderate COVID-19: the phase 2b part of a randomized, placebo-controlled, phase 2/3 study. *medRxiv* (2022) doi:10.1101/2022.06.22.22276792.

59. Mukae, H. *et al.* Efficacy and Safety of Ensitrelvir in Patients With Mild-to-Moderate Coronavirus Disease 2019: The Phase 2b Part of a Randomized, Placebo-Controlled, Phase 2/3 Study. *Clinical Infectious Diseases* **76**, 1403–1411 (2023).

60. Fahmi, O. A. *et al.* A combined model for predicting CYP3A4 clinical net drug-drug interaction based on CYP3a4 inhibition, inactivation, and induction determined in vitro. *Drug Metabolism and Disposition* **36**, 1698–1708 (2008).

Supplementary Figures

A)

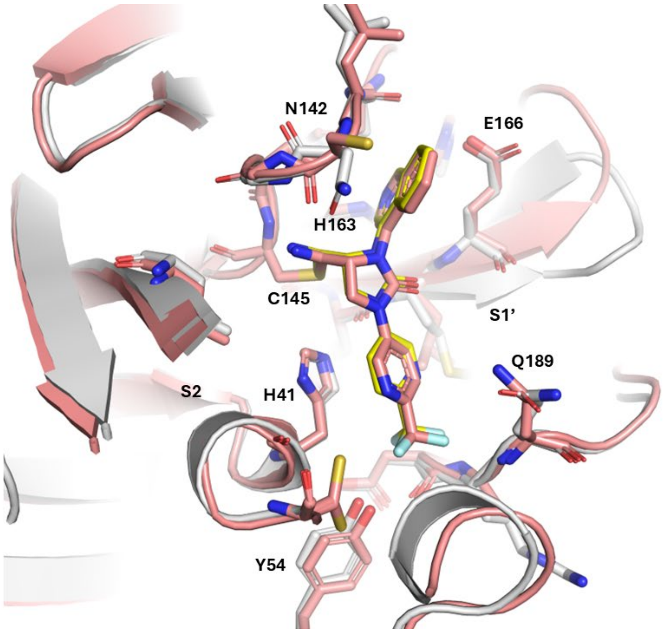

B)

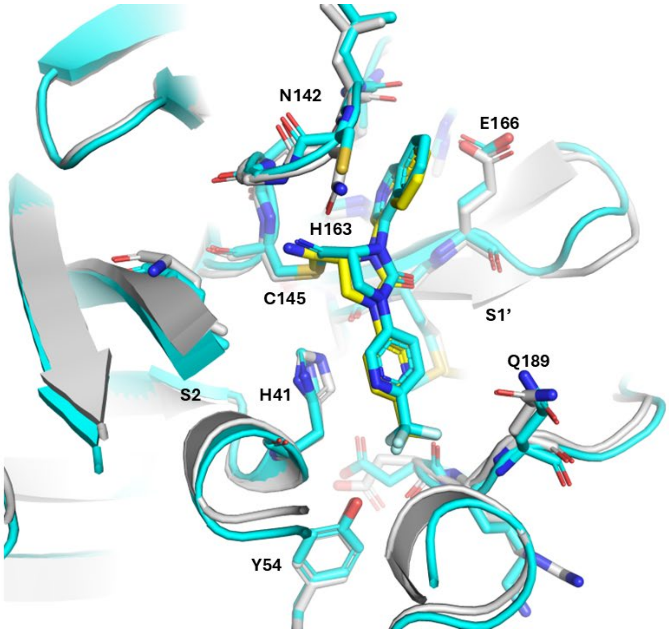

Figure S1. Structure of EGT710 in complex HCoV-OC43 Mpro and MERS-CoV Mpro. Crystal structure of EGT710 (yellow) in complex with SARS-CoV-2 Mpro (grey, PDB 9OIX) overlaid with structure of EGT710 in complex with A) HCoV-OC43 Mpro (pink, PDB 9Y8X) or B) MERS-CoV (blue, PDB 9Y8W). SARS-CoV-2 residues are numbered. Figures generated using PyMol software.

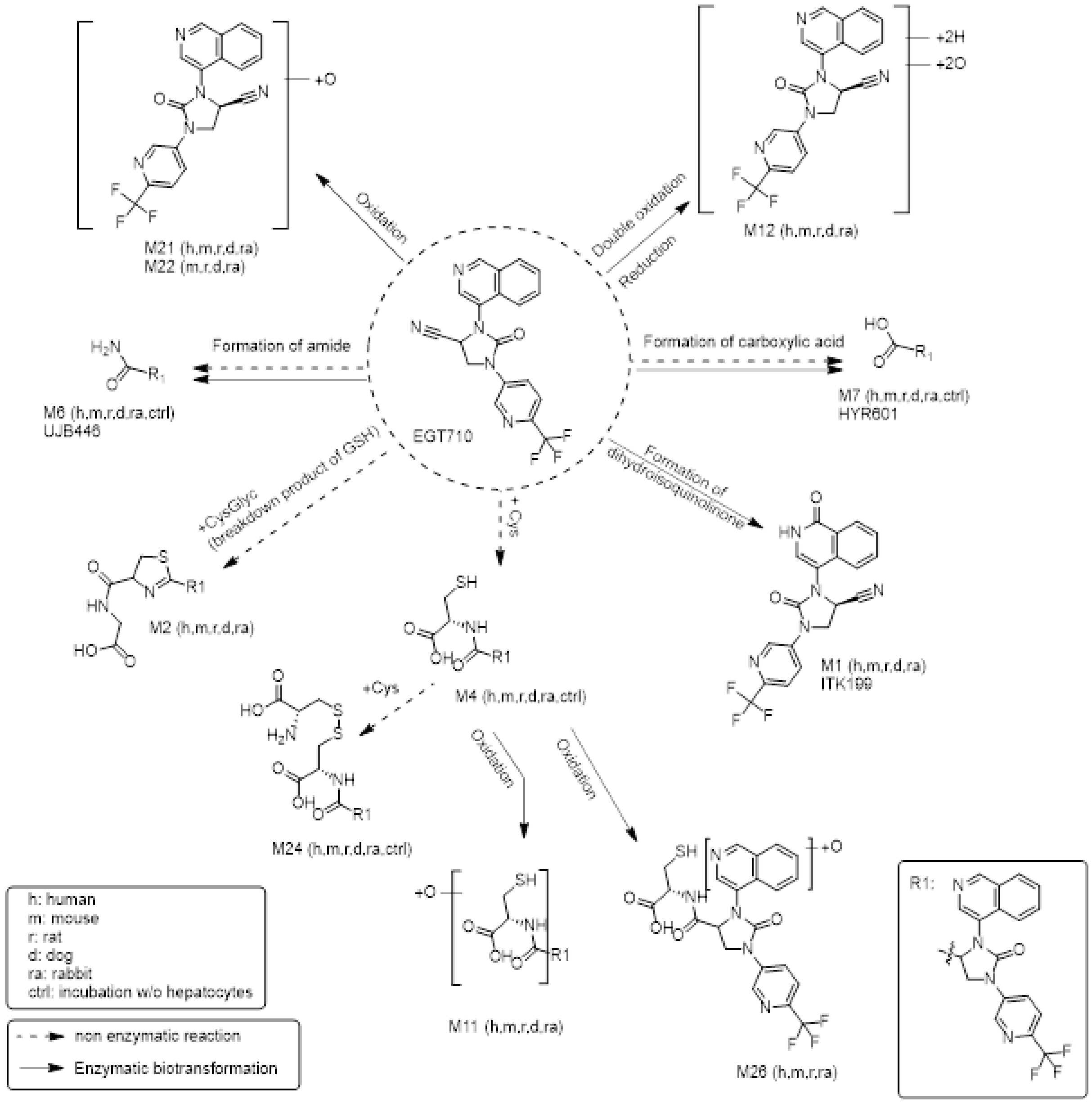

Figure S2: In vitro biotransformation pathways of EGT710 in hepatocytes of mouse, rat, rabbit, dog and human

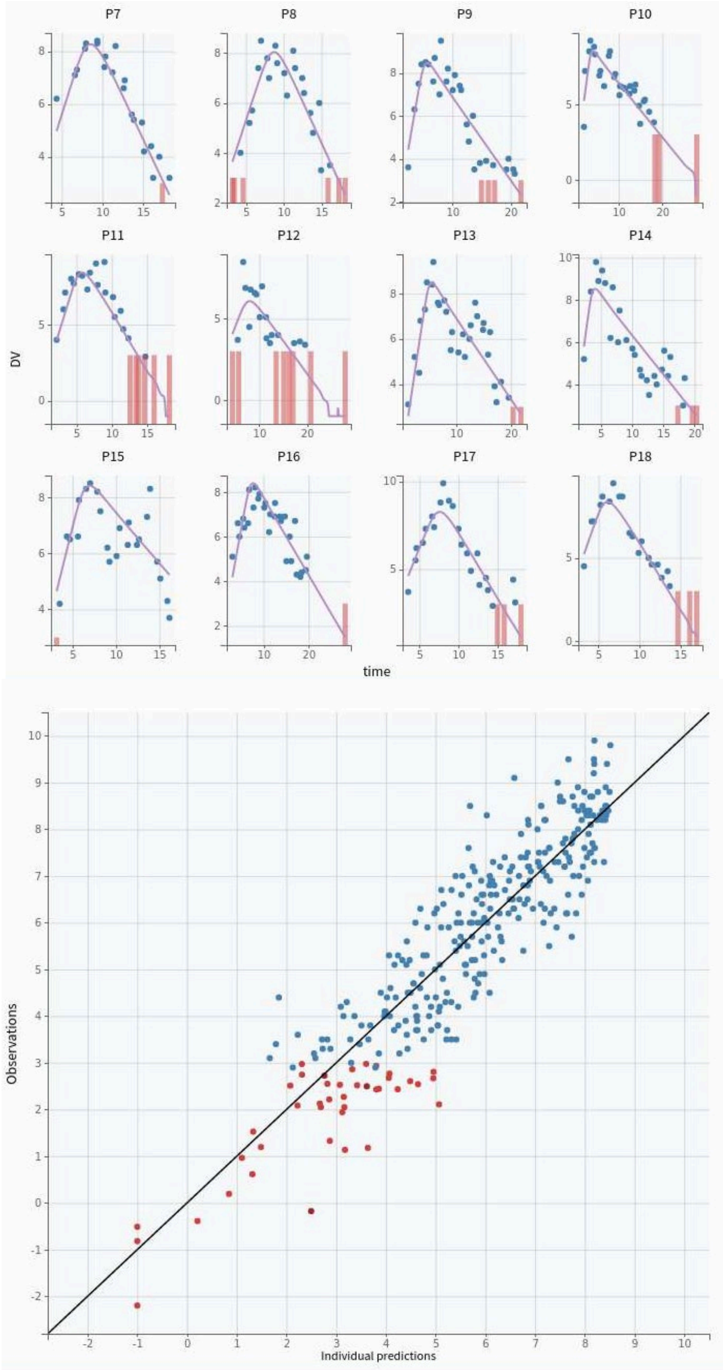

Figure S3. The one-state immune SARS-CoV-2 model recapitulates data digitized from [14], with subjects infected with the WT variant. (**Top**) Plots of viral loads (DV, expressed in log10 viral load (RNA copies/mL)) vs time (in days post-infection) for 12 subjects. Blue dots are digitized data, purple line model fit, red bars time-points where data were marked as censored for being below limit of detection (LOD) or lower limit of quantitation (LLOQ). (**Bottom**) Plot of predicted versus observed viral loads (expressed in log10 viral load (RNA copies/mL). Red points

are censored as being below LOD or LLOQ, and the reported observed value is that simulated by the model with the censoring constraints. Figures generated using Monolix software.

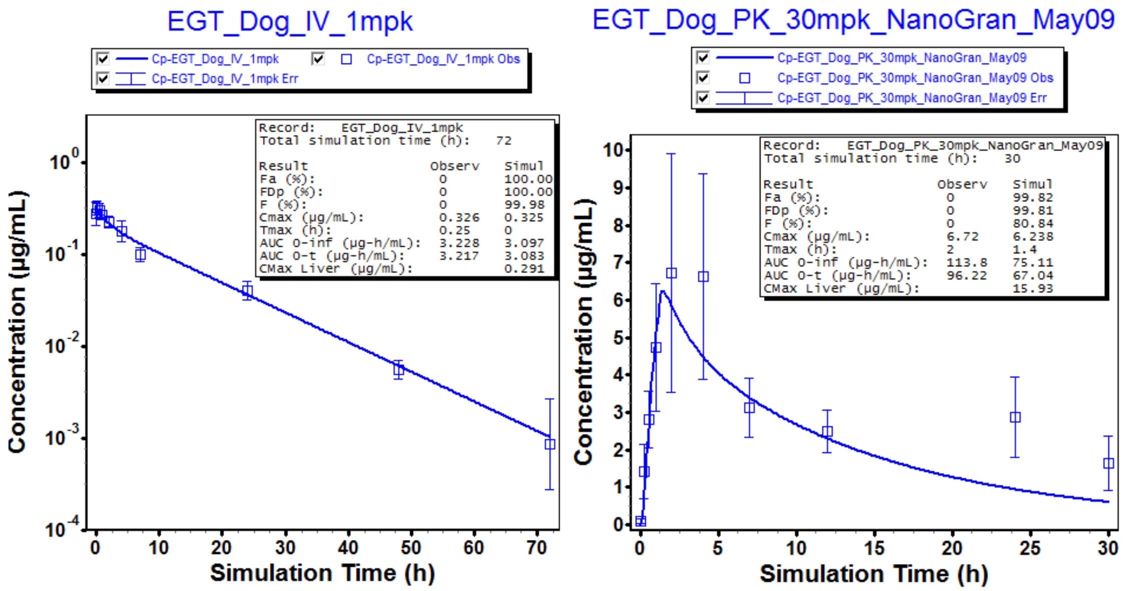

Figure S4. GastroPlus™-simulated vs observed dog EGT710 PK profiles for 1 mg/kg i.v. (left) and 30 mg/kg p.o. (as a nanosuspension). Figures generated using GastroPlus software.

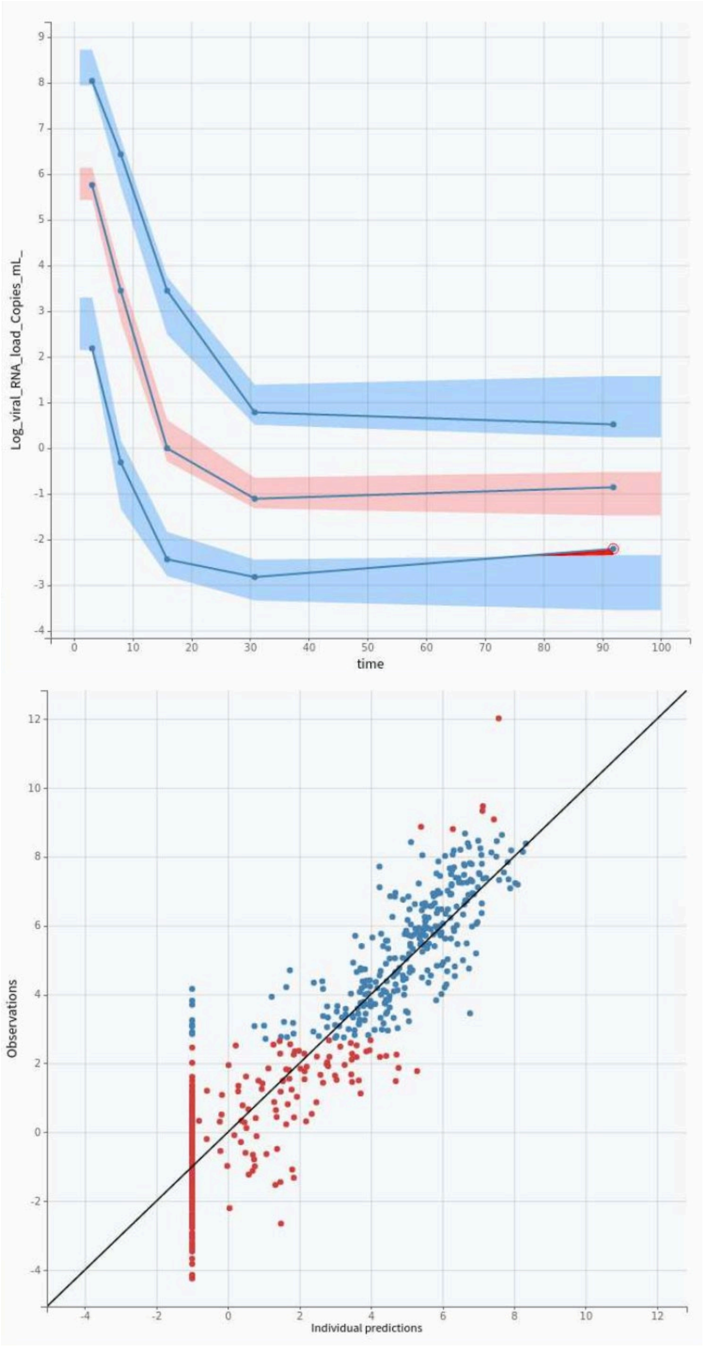

Figure S5. The one-state immune SARS-CoV-2 model recapitulates viral load data from the placebo arm of the EMPATHY study, with patients predominantly infected with the delta variant. (**Top**) Visual predictive check of model performance, where the Y-axis is a measure of log10 viral load (RNA copies/mL) and x-axis is time from diagnosis in days. The lower, middle, and upper solid blue lines represent the empirical 10th, 50th, and 90th percentiles respectively in the data across all individuals and the shaded regions represent the 90th prediction confidence interval around those percentiles. Red dots and dark red areas represent outliers. (**Bottom**) Plot of predicted versus observed viral loads (expressed in log10 viral load (RNA copies/mL). Red points are censored as being below LOD or LLOQ, and the reported observed value is that simulated by the model with the censoring constraints. Figures generated using Monolix software.

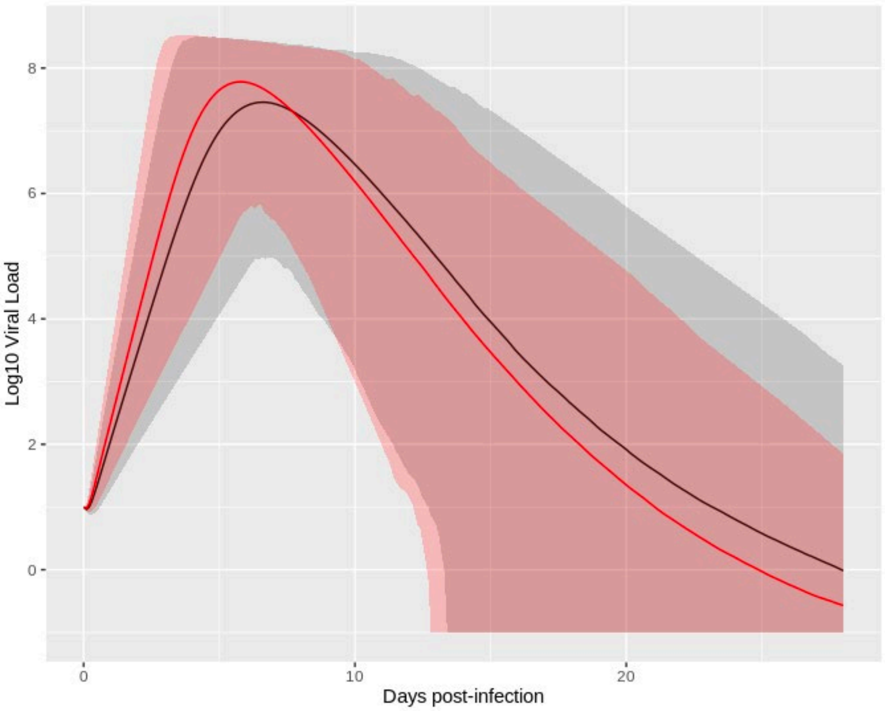

Figure S6. The population predictions of viral load kinetics by the WT and delta variant models broadly overlap. Plot of log10 viral load versus time (in days post infection) for the WT variant model (black) and delta variant model (red). Mean (solid line), +/- 95% prediction interval (shaded regions). Figures generated using R software.

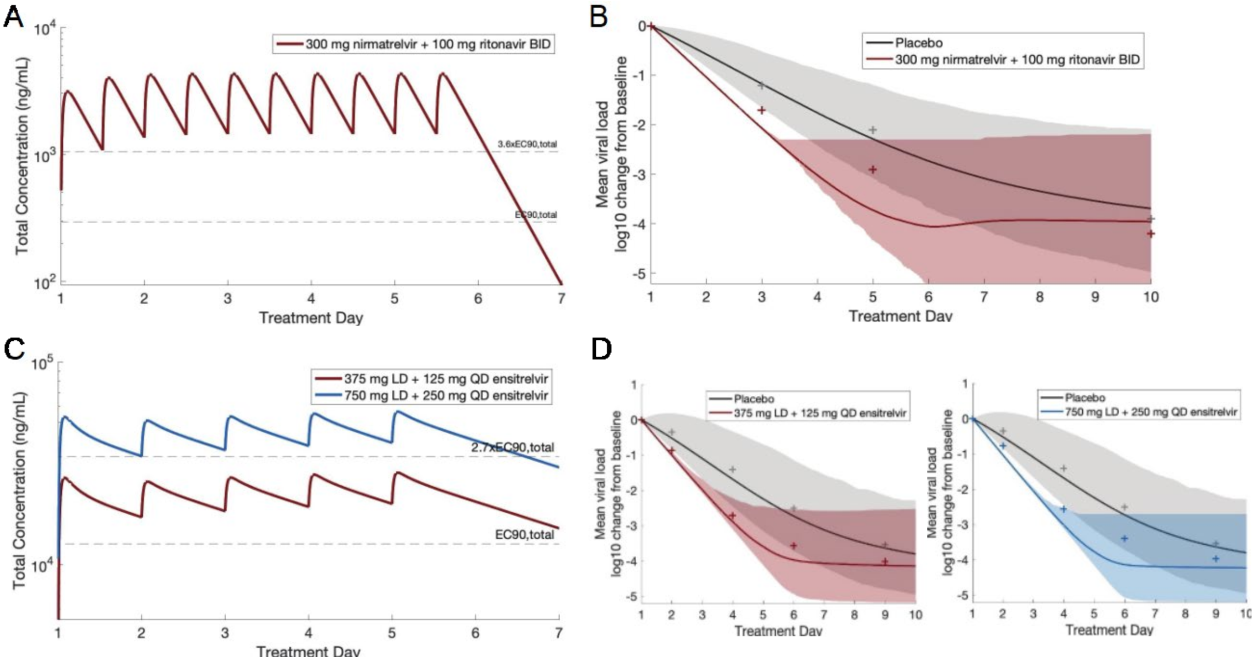

Figure S7. The SARS-CoV-2 delta variant viral kinetics model can reproduce the reported viral loads following treatment with placebo, nirmatrelvir+ritonavir, or ensitrelvir. (**A**) Plot of simulated nirmatrelvir PK following administration of 300 mg nirmatrelvir + 100 mg ritonavir. (**B**) Plot of simulated (mean, solid lines; 95% prediction intervals, shaded areas) viral loads vs reported (+; [13]) for placebo (black/gray) and 300 mg nirmatrelvir + 100 mg ritonavir (red). (**C**) Plot of simulated nirmatrelvir PK following administration of 375 mg loading dose + 125 mg QD ensitrelvir (red) or 750 mg loading dose + 250 mg QD ensitrelvir. (**D**) Plots of simulated (mean, solid lines; 95% prediction intervals, shaded areas) viral loads vs reported (+; [57]) for placebo (black/gray) and 300 mg nirmatrelvir + 100 mg ritonavir (red) or 750 mg loading dose + 250 mg QD ensitrelvir. Figures generated using Matlab software.

Table S1. In vitro activity of EGT710 against various coronavirus Mpro enzymes. Limit of detection for the assay (lowest compound concentration used) was 0.0025 μM based on the enzyme concentration used in the assay (0.005 μM)

Coronavirus genus	Mpro enzyme	EGT710 IC_{50} (μM) [μg/mL][a]
Alphacoronavirus	HCoV-229E	<0.0025 [<0.0010][b]
	HCoV-NL63	0.0038 [0.0015][b]
Betacoronavirus	HCoV-OC43	0.011 [0.0043][b]
	HCoV-HKU1	0.0035 [0.0013][c]
	SARS-CoV-2	<0.0025 [<0.0010][b]
	SARS-CoV	<0.0025 [<0.0010][b]
Deltacoronavirus	PorCoV-HKU15	0.068 [0.026][b]
Gammacoronavirus	IBV	0.297 [0.114][b]

Table S2. Additional mutations found in passaged viruses. Mutations with a prevalence above 10% are listed, and mutations in the mNG gene are excluded

Sel-1

Position	Ref	Alt	Fraction	Amino acid
3737	C	T	10.26%	NSP3, P340S
10202	C	T	99.92%	NSP5, L50F
10551	A	C	99.96%	NSP5, E166A
12060	A	C	92.83%	NSP7, E73A
16916	T	C	99.96%	NSP13, L227P
20566	A	G	99.97%	NSP15, K316E
21752	T	A	10.87%	Spike, W64R
23934	C	T	40.35%	Spike, T791I
28652	C	T	99.97%	3'UTR

Sel-2

Position	Ref	Alt	Fraction	Amino acid
147	C	T	96.28%	5'UTR
866	A	G	17.33%	NSP2, I21V
4784	C	T	19.22%	NSP3, L689F
6976	T	G	75.47%	NSP3, S1419R
10116	C	T	69.83%	NSP5, T21I
10202	C	T	99.90%	NSP5, L50F
10626	C	T	29.80%	NSP5, A191V
12020	C	T	19.99%	NSP7, L60F
12499	C	A	67.07%	NSP8, N136K
16778	T	C	86.97%	NSP13, V181A
16916	T	C	13.94%	NSP13, L227P
18928	C	T	31.61%	NSP14, P297S
19397	A	T	20.11%	NSP14, E453V
20637	A	AGAAGATTTT	64.27%	NSP15, E339EEDF
20930	C	T	13.01%	NSP16, T91M
23525	C	T	17.31%	Spike,H655Y
28571	GAGTGTAC	G	11.99%	3'UTR deletion

Sel-3

Position	Ref	Alt	Fraction	Amino acid
10202	C	T	99.95%	NSP5, L50F
11403	A	C	99.94%	NSP6, N144T
11750	C	T	99.96%	NSP6, L260F
16388	A	G	27.48%	NSP13, N51S
16778	T	C	99.90%	NSP13, V181A

Sel-4

Position	Ref	Alt	Fraction	Amino acid
2883	G	T	45.58%	NSP3, C55F
3236	C	G	33.57%	NSP3, Q173E
5607	A	G	26.60%	NSP3, K963R
5907	C	T	52.78%	NSP3, T1063I
6293	A	G	30.43%	NSP3, I1192V
10202	C	T	99.95%	NSP5, L50F
10691	A	G	36.48%	NSP5, I213V
11511	C	T	99.88%	NSP6, T180I
15251	C	T	100.00%	NSP12, T596I
17221	A	G	99.91%	NSP13, K329E
19682	A	G	53.30%	NSP15, E21G
13783	T	G	40.21%	NSP12, S107A
24450	T	C	57.53%	Spike, V963A
28528	A	G	99.86%	3'UTR
28541	A	AT	76.40%	3'UTR

Table S3. EGT710 as in vitro inhibitor or inducer of enzymes or transporters

Affected protein [a)]	Kinetic parameter (Inhibition: Ki, IC50 Induction: EC50, Emax)	DDI assessment and potential clinical implications [b)]
DDI risk concerning enzymes in the liver and in the intestine		
CYP1A2	EC50: 36.0 µM<br>Emax: 16.3	In vitro, EGT710 was a reversible inhibitor of CYP2C8 and CYP3A4/5, and an inducer of CYP1A2, CYP2B6, CYP2C8, CYP2C9 and CYP3A4/5.<br>Based on PBPK modeling of DDI magnitude moderate induction of CYP3A4 (AUC ratio of 0.35) and weak induction of CYP2C8 (AUC ratio of 0.68) was predicted with midazolam and repaglinide as sensitive substrates, respectively. No clinically relevant induction of CYP1A2 was anticipated. Based on static DDI assessment[b,c], EGT710 may also be a moderate inducer of CYP2B6 and CYP2C9.
CYP2B6	EC50: 0.19 µM<br>Emax: 2.5	
CYP2C8	Ki,u: 50.5 µM<br>EC50: 1.1 µM<br>Emax: 6.5	
CYP2C9	EC50: 0.31 µM<br>Emax: 3.3	
CYP3A4/5	Ki,u: 33.3 µM<br>EC50: 1.6 µM<br>Emax: 13.4	
DDI risk concerning solute-carrier transporters at the liver inlet		
OATP1B1	Ki: 65.6 µM	The weak in vitro inhibition potential of EGT710 towards OATP1B1, OAPT1B3 and OCT1 is unlikely to translate into clinical significance as the experimentally determined inhibition constants are more than 30-fold higher than Ch,in,u.
OATP1B3	Ki: 51.6 µM	
OCT1	Ki: 77.8 µM	
DDI risk concerning solute-carriers transporters in the kidney		
OAT1	Ki: 39.9 µM	In vitro, EGT710 was an inhibitor of renal transporters. Based on static DDI assessment[c] using a predicted systemic maximal unbound concentration (Cmax,u) of 0.73 µM[b], EGT710 may inhibit MATE2-K at therapeutic concentrations. Inhibition of OAT3 and MATE1 cannot be excluded.
OAT3	Ki: 9.56 µM	
OCT2	Ki: 68.7 µM	
MATE1	Ki: 13.8 µM	
MATE2-K	Ki: 1.79 µM	
DDI risk concerning ubiquitous efflux transporters (liver/kidney, intestine, etc.)		
MDR1	Ki: 86.4 µM	In vitro, EGT710 was an inhibitor of efflux transporters MDR1 and BSEP. EGT710 is not expected to inhibit systemic MDR1 and BSEP since the Ki values are more than 80-fold higher than Cmax,u. Dynamic DDI simulation indicates weak inhibition of intestinal MDR1 (AUC ratio of 1.24) by EGT710.
BSEP	Ki: 60.6 µM	

a. Additional activities tested but not affected: reversible inhibition of CYP1A2, CYP2A6, CYP2B6, CYP2C9, CYP2C19, CYP2D6 and CYP2E1 (tested up to 100 µM), time-dependent inhibition of CYP1A2, CYP2A6, CYP2B6, CYP2C8, CYP2C9, CYP2C19, CYP2D6, CYP2E1 and CYP3A4/5 (tested up to 100 µM), inhibition of BCRP up to 60 µM and MRP2 up to 125 µM.

b. DDI risk calculations for EGT710 after multiple oral doses of 600 mg QD for 7 days were done using a maximal unbound concentration (Cmax,u) of 0.73 µM (280 ng/mL) calculated with an extrapolated Cmax,total of 6.73 µM (2577 ng/mL) and fu at 1 µM of 0.109, an estimated maximal unbound hepatic inlet concentration (Ch,in,u) of about 1.57 µM, an anticipated maximal intestinal gut lumen concentration of 6.28 mM and a projected maximal enterocyte concentration (Cgut,ent) of 50 µM.

c. Static net effect assessment according to [60]

Table S4. Summary of predicted dose regimen across various conditions to achieve various EC_{90} targets. HFHC, high fat high carbohydrate

Scenario	EC_{90} target	QD (mg)
Fasted	1x	190
	2x	360
	3x	700
Fed (HFHC meal)	1x	180
	2x	360
	3x	540

Table S5. Activity of EGT710 against human proteases.

Cat #	Assay Name	Batch*	Spec.	Rep.	Conc.	% Inh.	IC_{50}*	K_i	n_H	R
163100	Peptidase, CASP2 (Caspase 2)	478147	hum	1	10 µM	-4				
			hum	1	1 µM	-1				
163200	Peptidase, CASP3 (Caspase 3)	478148	hum	1	10 µM	-10				
			hum	1	1 µM	-5				
163400	Peptidase, CASP7 (Caspase 7)	478152	hum	1	10 µM	15				
			hum	1	1 µM	3				
163500	Peptidase, CASP8 (Caspase 8)	478153	hum	1	10 µM	0				
			hum	1	1 µM	5				
113400	Peptidase, Chymotrypsin	478324	hum	1	10 µM	4				
			hum	1	1 µM	7				
112250	Peptidase, CTSB (Cathepsin B)	478320	hum	1	10 µM	-18				
			hum	1	1 µM	-1				
112350	Peptidase, CTSD (Cathepsin D)	478138	hum	1	10 µM	1				
			hum	1	1 µM	-15				
112400	Peptidase, CTSE (Cathepsin E)	478139	hum	1	10 µM	-16				
			hum	1	1 µM	-12				
112600	Peptidase, CTSK (Cathepsin K)	478141	hum	1	10 µM	6				
			hum	1	1 µM	9				
112650	Peptidase, CTSL (Cathepsin L)	478144	hum	1	10 µM	0				
			hum	1	1 µM	1				
112750	Peptidase, CTSS (Cathepsin S)	478145	hum	1	10 µM	-11				
			hum	1	1 µM	0				
166010	Peptidase, ELA2 (Neutrophil Elastase 2)	478328	hum	1	10 µM	0				
			hum	1	1 µM	-2				
163950	Peptidase, Endothelin Converting Enzyme-1 (ECE-1)	478331	hum	1	10 µM	-8				
			hum	1	1 µM	-5				
113500	Peptidase, Factor VIIa	478313	hum	1	10 µM	5				
			hum	1	1 µM	4				
165000	Peptidase, Thrombin	478311	hum	1	10 µM	-8				
			hum	1	1 µM	-9				
165200	Peptidase, Tryptase	478327	hum	1	10 µM	-5				
			hum	1	1 µM	-8				

Note: Items meeting criteria for significance (≥50% stimulation or inhibition) are highlighted.
* Batch: Represents compounds tested concurrently in the same assay(s).
hum=Human

Table S6. Activity of EGT710 against a panel of ion-channels, transporters, enzymes, GPCRs etc. Target species include: human (h), rat (r), porcine (p). Abbreviations: GPCR, G-Protein Coupled Receptor; NHR, Nuclear Hormone Receptor, * % stimulation 10µM.

					Potency	
**Target name**	**Gene symbol**	**Class**	**Mode**	**Format**	**IC_{50} (µM)**	**% inhibition 10 µM**
A_1 receptor (h)	ADORA1	GPCR	Inhibition	Binding	>30	
A_{2A} receptor (h)	ADORA2A	GPCR	Inhibition	Binding		3
A_3 receptor (h)	ADORA3	GPCR	Inhibition	Binding		11
α_{1A}-adrenoceptor (h)	ADRA1A	GPCR	Inhibition	Binding	>30	
α_{2A}-adrenoceptor (h)	ADRA2A	GPCR	Inhibition	Binding	>30	
α_{2B}-adrenoceptor (h)	ADRA2B	GPCR	Inhibition	Binding		7
β_1-adrenoceptor (h)	ADRB1	GPCR	Inhibition	Binding		-2
β_2-adrenoceptor (h)	ADRB2	GPCR	Inhibition	Binding		
β_3-adrenoceptor (h)	ADRB3	GPCR	Inhibition	Binding		-11
apelin receptor (h)	APLNR	GPCR	Inhibition	Binding		-5
BB_2 receptor (h)	GRPR	GPCR	Inhibition	Binding		11
CB_1 receptor (h)	CNR1	GPCR	Antagonism	Functional		-5
CCK_2 receptor (h)	CCKBR	GPCR	Inhibition	Binding		-1
D_1 receptor (h)	DRD1	GPCR	Inhibition	Binding	>30	
D_2 receptor (h)	DRD2	GPCR	Inhibition	Binding		21
D_3 receptor (h)	DRD3	GPCR	Inhibition	Binding	>30	
ET_A receptor (h)	EDNRA	GPCR	Inhibition	Binding		-3
ghrelin receptor (h)	GHSR	GPCR	Inhibition	Binding		-38
H_1 receptor (h)	HRH1	GPCR	Agonism	Functional		1*
H_3 receptor (h)	HRH3	GPCR	Inhibition	Binding	>30	
Imidazoline receptor (r)	MAOA	GPCR	Inhibition	Binding		-30
$CysLT_1$ receptor (h)	CYSLTR1	GPCR	Inhibition	Binding		-4
$CysLT_2$ receptor (h)	CYSLTR2	GPCR	Inhibition	Binding		3
MC_1 receptor (h)	MC1R	GPCR	Inhibition	Binding		-9
MT_1 receptor (h)	MTNR1A	GPCR	Inhibition	Binding		-7
MT_2 receptor (h)	MTNR1B	GPCR	Inhibition	Binding		9
motilin receptor (h)	MLNR	GPCR	Inhibition	Binding		-2
M_1 receptor (h)	CHRM1	GPCR	Inhibition	Binding	>30	
M_2 receptor (h)	CHRM2	GPCR	Inhibition	Binding	>30	
M_3 receptor (h)	CHRM3	GPCR	Inhibition	Binding		4
NTS_1 receptor (h)	NTSR1	GPCR	Inhibition	Binding		4
δ receptor (h)	OPRD1	GPCR	Inhibition	Binding	>30	
µ receptor (h)	OPRM1	GPCR	Inhibition	Binding	>30	
OX_1 receptor	HCRTR1	GPCR	Inhibition	Binding		29
OT receptor (h)	OXTR	GPCR	Inhibition	Binding		1
PAF receptor (h)	PTAFR	GPCR	Inhibition	Binding		4
DP_1 receptor (h)	PTGDR1	GPCR	Inhibition	Binding		1
DP_2 receptor (h)	PTGDR2	GPCR	Inhibition	Binding		9
EP_1 receptor (h)	PTGER1	GPCR	Inhibition	Binding		-14
EP_2 receptor (h)	PTGER2	GPCR	Inhibition	Binding		3

Target name	Gene symbol	Class	Mode	Format	Potency	
					IC_{50} (μM)	% inhibition 10 μM
EP_3 receptor (h)	PTGER3	GPCR	Inhibition	Binding		8
EP_4 receptor (h)	PTGER4	GPCR	Inhibition	Binding		-2
FP receptor (h)	PTGFR	GPCR	Inhibition	Binding		-9
IP receptor (h)	PTGIR	GPCR	Inhibition	Binding		-9
5-HT_{1A} receptor (h)	HTR1A	GPCR	Inhibition	Binding	>30	
5-HT_{1B} receptor (h)	HTR1B	GPCR	Inhibition	Binding		-19
5-HT_{2A} receptor (h)	HTR2A	GPCR	Inhibition	Binding		-9
5-HT_{2B} receptor (h)	HTR2B	GPCR	Agonism	Functional	>30*	
5-HT_{2C} receptor (h)	HTR2C	GPCR	Inhibition	Binding		4
5-HT_3 receptor (h)	HTR3A	GPCR	Inhibition	Binding		-10
5-HT_4 receptor (h)	HTR4	GPCR	Inhibition	Binding		-15
5-HT_6 receptor (h)	HTR6	GPCR	Inhibition	Binding		6
5-HT_7 receptor (h)	HTR7	GPCR	Inhibition	Binding		-5
Sigma non-opioid intracellular receptor 1 (h)	SIGMAR1	GPCR	Inhibition	Binding		19
sst_1 receptor (h)	SSTR1	GPCR	Inhibition	Binding		-4
sst_3 receptor (h)	SSTR3	GPCR	Inhibition	Binding		-14
sst_5 receptor (h)	SSTR5	GPCR	Inhibition	Binding		0
TP receptor (h)	TBXA2R	GPCR	Agonism	Functional	>30*	
TP receptor (h)	TBXA2R	GPCR	Antagonism	Functional	>30	
UT receptor (h)	UTS2R	GPCR	Inhibition	Binding		-5
V_{1A} receptor (h)	AVPR1A	GPCR	Inhibition	Binding		-15
Androgen receptor (h)	AR	NHR	Inhibition	Binding	>30	
Estrogen alpha receptor (h)	ESR1	NHR	Inhibition	Binding	>30	
Glucocorticoid receptor (h)	NR3C1	NHR	Inhibition	Binding		-15
PPARgamma receptor (h)	PPARG	NHR	Inhibition	Binding		17
Progesterone receptor (h)	PGR	NHR	Inhibition	Binding	>30	
Vitamin D3 receptor (h)	VDR	NHR	Inhibition	Binding		-7
$Ca_v1.2$ (h)	CACNA1C	Ion Channel	Inhibition	Patch Clamp	>50	
$Ca_v2.2$ (r)	CACNA1B	Ion Channel	Inhibition	Binding		-22
$Na_v1.5$ (h)	SCN5A	Ion Channel	Inhibition	Patch Clamp	>50	
GABA A chloride channel (r)	GABRA1	Ion Channel	Inhibition	Binding		-1
Glutamate (AMPA) (r)	GRIA3	Ion Channel	Inhibition	Binding		14
Glutamate (NMDA, agonism) (r)	GRIN1	Ion Channel	Inhibition	Binding		1
Glutamate (NMDA, PCP) (r)	GRIN1	Ion Channel	Inhibition	Binding		3
Glutamate (NMDA, polyamine) (r)	GRIN1	Ion Channel	Inhibition	Binding		13
Glycine (strychnine-sensitive) (r)	GLRA1	Ion Channel	Inhibition	Binding		7
hERG (h)	KCNH2	Ion Channel	Inhibition	Binding	>30	
hERG (h)	KCNH2	Ion Channel	Inhibition	Patch Clamp	>30	
nicotinic acetylcholine $\alpha_4\beta_2$ ion channel (h)	CHRNB2	Ion Channel	Inhibition	Binding		-23
RyR2 (h)	RYR2	Ion Channel	Inhibition	Binding		1

Target name	Gene symbol	Class	Mode	Format	Potency	
					IC$_{50}$ (μM)	% inhibition 10 μM
Benzodiazepine Site (Central) (r)	GABRA1	Ion Channel	Inhibition	Binding	>30	
ENT1 (h)	SLC29A1	Transporter	Inhibition	Binding		-8
BSEP (h)	ABCB11	Transporter	Uptake	Functional	32	
CHT (h)	SLC5A7	Transporter	Inhibition	Binding		-1
DAT (h)	SLC6A3	Transporter	Inhibition	Binding	>30	
GAT1 (h)	SLC6A1	Transporter	Inhibition	Binding		3
NET (h)	SLC6A2	Transporter	Inhibition	Binding	>30	
SERT (h)	SLC6A4	Transporter	Inhibition	Binding	>30	
VMAT2 (r)	SLC18A2	Transporter	Uptake	Functional	19	
Acetylcholinesterase (h)	ACHE	Enzyme	Inhibition	Enzymatic	>30	
5-Lipoxygenase (h)	ALOX5	Enzyme	Inhibition	Enzymatic		-10
Caspase 3 (h)	CASP3	Enzyme	Inhibition	Enzymatic		-4
Cathepsin D (h)	CTSD	Enzyme	Inhibition	Enzymatic		-35
Cyclooxygenase 1 (h)	PTGS1	Enzyme	Inhibition	Enzymatic	>30	
Cyclooxygenase 2 (h)	PTGS2	Enzyme	Inhibition	Enzymatic	>30	
Sirtuin 6 (h)	SIRT6	Enzyme	Inhibition	Enzymatic		2
Matrix Metalloproteinase 8 (h)	MMP8	Enzyme	Inhibition	Enzymatic		-7
Monoamine Oxidase A (h)	MAOA	Enzyme	Inhibition	Enzymatic	>30	
Monoamine Oxidase B (h)	MAOB	Enzyme	Inhibition	Enzymatic		39
Phosphodiesterase 3A (h)	PDE3A	Enzyme	Inhibition	Enzymatic	>30	
Phosphodiesterase 4D (h)	PDE4D	Enzyme	Inhibition	Enzymatic	>30	
Protein tyrosine phosphatase (PTEN) (h)	PTEN	Enzyme	Inhibition	Enzymatic		0
Protein tyrosine phosphatase (PTPN6) (h)	PTPN6	Enzyme	Inhibition	Enzymatic		4
ATPase, Na+/K+ (p)	ATP1A2	Enzyme	Inhibition	Enzymatic		5
Thrombin (h)	F2	Enzyme	Inhibition	Enzymatic	>30	

Table S7. Agilent RapidFire method parameters

Step	Time (ms)					
Aspirate	700					
Load	3000					
Extra Wash	0					
Elute	6000					
Re-equilibration	1500					
Mass spectrometer MRM settings						
Mpro Enzyme	Analyte (charge state)	Q1 (m/z)	Q3 (m/z)	Dwell time (ms)	CE (V)	DP (V)
HCoV-229E	Product (1+)	448.1	260.2	10	25	56
HCoV-229E	Substrate (2+)	552.9	675.1	10	35	51
HCoV-NL63	Product (1+)	448.1	260.2	10	25	50
HCoV-NL63	Substrate (2+)	546.9	663.2	10	33	86
HCoV-OC43	Product (1+)	494.2	260.2	10	25	56
HCoV-OC43	Substrate (2+)	555.4	235.1	10	29	76
HCoV-HKU1	Product (1+)	494.2	260.2	10	25	56
HCoV-HKU1	Substrate (2+)	555.4	235.1	10	29	76
SARS-CoV-2	Product (1+)	430.3	260.4	10	18	100
SARS-CoV-2	Substrate (3+)	378.6	482.6	10	23	80
SARS-CoV	Product (1+)	430.3	260.4	10	18	100
SARS-CoV	Substrate (3+)	378.6	482.6	10	23	80
PorCoV-HKU15	Product (1+)	489.2	260.1	10	31	101
PorCoV-HKU15	Substrate (2+)	599.6	533.9	10	29	96
IBV	Product (1+)	503.2	227.1	10	43	146
IBV	Substrate (2+)	574.3	888.2	10	33	151
Mass spectrometer settings						
Parameter	Value	Unit				
Temperature	650	˚C				
Curtain Gas	40	psi				
Collision Gas	9	-				
IonSpray Voltage	5500	V				
Ion Source Gas 1	60	psi				
Ion Source Gas 2	60	psi				

CE, collision energy; DP, declustering potential; m/z, mass/charge number of ions; PorCoV-HKU15, porcine coronavirus HKU15; psi, pounds per square inch

Table S8. RapidFire-mass spectrometry assay reaction conditions

Mpro enzyme	Substrate	Product	Concentration (μM)		Incubation time (hours)
			Enzyme	Substrate	
HCoV-229E	STLQAGLRKM	STLQ	10	10	3.0
HCoV-NL63	STLQSGLKKM	STLQ	10	10	3.0
HCoV-OC43	SFLQSGIVKM	SFLQ	5	5	2.0
HCoV-HKU1	SFLQSGIVKM	SFLQ	5	5	2.0
SARS-CoV-2	AVLQSGFRKM	AVLQ	5	5	2.0
SARS-CoV	AVLQSGFRKM	AVLQ	5	5	2.5
PorCoV-HKU15	TKLQAGIKILL	TKLQ	5	10	2.5
IBV	SRLQAGFKKL	SRLQ	5	10	2.5

Table S9. Predicted human total clearance (CL) according to different allometric scaling methods

**Method**	**Predicted human clearance (mL/min/kg)**	**Slope**	**ROE**
Simple allometry without fu	4.9	0.99	No
Simple allometry with fu	2.6	0.87	
Product of maximum life span potential and clearance	1.7	1.31	Yes (exponent:0.97)
Product of brain weight and clearance	1.1	1.86	No
CL-NAS-hep (incorporation of in vitro microsome data)	4.5	1.01	
CL-NAS-mic (incorporation of in vitro microsome data)	2.8	0.92	
Fu corrected intercept method	1.5		
Single-species allometric scaling (dog)	2.7		
Single-species allometric scaling (mouse)	0.8		
Single-species allometric scaling (rat)	1.0		
Two species proportionality using bound drug (rat-dog-human)	0.9	1.01	
CL-IVIVE from microsomes	2.7		
CL-IVIVE from hepatocytes	2.3		
**Average (Standard deviation)**	**2.2 (1.2)**		

Table S10. Predicted human volume of distribution (Vss) according to different allometric scaling methods

Method	Predicted human Vss (L/kg)	Slope
Simple allometry without fu	4.19	1.15
Simple allometry with fu	2.71	1.05
Oiz-Tozer equation	1.97	
Single-species allometric scaling (mouse)	1.30	
Single-species allometric scaling (rat)	1.78	
Single-species allometric scaling (dog)	3.16	
Single-species allometric scaling with fu (mouse)	1.89	
Single-species allometric scaling with fu (rat)	1.78	
Single-species allometric scaling with fu (dog)	2.55	
Two species scaling (rat-dog to human)	1.84	
**Average (Standard deviation)**	**2.32 (0.86)**	

Table S11. Parameters for 2-compartment PK model fit to the Wajima-predicted human i.v. systemic concentration-time profile for 1 mg/kg EGT710

Parameter	Value
Bodyweight	70 kg
V_1 (L/kg)	1.55
V_2 (L/kg)	0.90
CL (L/h/kg)	0.13
k_{12} (1/h)	0.138
k_{21} (1/h)	0.237
$T_{1/2}$ (h)	13.95

Table S12. Input parameters for GastroPlus™ human PBPK model for EGT710

Parameter		Value		Source
**Compound, formulation and ACAT model**				
MW (g/mol)		383.33		
logD pH 7.4		2.4		Measured
Measured pKa		4.2 (b)		Measured
Papp MDCK-LE v2 [x10^{-6}] cm/s		21.4		Measured
Peff [x10^{-4}] cm/s		Human<br>3.66		Human Peff calculated based on MDCK-LE V2
	**Medium pH**	**Solubility (µg/mL)**	**Final pH**	
Thermodynamic solubility NX Type IV polymorph	aq. pH 6.5 [a]	10	6.8	Measured
	SGF (pH 1.6)	690	1.7	
	FaSSIF V1 (pH 6.5)	14	6.6	
	FeSSIF V2 (pH 6.8)	28	6.7	
Other solubility	2X FeSSIF pH 6.8 [b]	49	6.8	Measured
Fitted pKa		3.518 (SF 199.3)		Estimated in GastroPlus
Dissolution model		Johnson dissolution model		GastroPlus
Particle radius (um)		0.1		Nanosuspension
Bile salt solubilisation ratio		11 500 (based on FaSSIF-V1 and FeSSIF-V2)		Estimated in GastroPlus
Precipitation time (s)		900		Assumed GastroPlus default
Diffusion coefficient (cm^2/s x 10^5)		0.72		ADMET predictor (GastroPlus)
Dosage form		IR Capsule		-
Administration volume, human (mL)		250		-
Additional liver FPE		15%		Assumed
Gastrointestinal fluid volumes (Virtual physiology)		40 % small intestine, 10 % colon		GastroPlus default
Absorption scale factor (ASF) model		Opt logD Model SA/V 6.1		GastroPlus default
**PK model: Compartmental model**				
Bodyweight		70 kg		-
V1 (L/kg)		1.55		Wajima
V2 (L/kg)		0.90		Wajima
CL (mL/min/kg)		2.22		Allometry
k12 (1/h)		0.138		Wajima
k21 (1/h)		0.237		Wajima
B/P ratio		1.21		Measured
Plasma Fup (%)[c]		10.9		Measured

[a] Reference solubility; [b] 'User' solubility, with 20 mM bile salts, in human model; Plasma Fup (%) was measured at 1 µM EGT710 (10.9%) and 10 µM EGT710 (12.7%). The value for plasma Fup (%), as measured at 1µM EGT710 is used, as it is closer to the 3XEC90,total threshold relevant to dose prediction (1.75 µM).

Table S13. Summary of model parameters for the WT SARS-CoV-2 variant

Parameter	Units	Value	Standard Error (Relative standard error)
**Fixed effects**			
beta	1/day	54.6	6.92 (12.7%)
delta	1/day	0.82	0.00034 (0.0408%)
p	1/day	0.39	0.00054 (0.137%)
c	1/day	2.4	0.0043 (0.177%)
epsilon	1/(nanomole/liter)	1.76	0.0095 (0.539%)
eta	1/(nanomole/liter)	0.37	0.51 (138%)
**Standard deviation of the random effects**			
Omega_beta	1/day	0.43	0.093 (21.7%)
Omega_eta	1/(nanomole/liter)	4.27	1.16 (27.3%)
**Error model parameters**			
a		0.96	0.043 (4.46%)

Table S14. Summary of model parameters for the SARS-CoV-2 delta variant

Parameter	Units	Value	Standard Error (Relative standard error)
**Fixed effects**			
beta	1/day	71.32	5.89 (8.26%)
delta	1/day	0.82	n.e.
p	1/day	0.39	n.e.
c	1/day	2.4	n.e.
epsilon	1/(nanomole/liter)	1.76	n.e.
eta	1/(nanomole/liter)	0.42	0.14 (32.7%)
**Standard deviation of the random effects**			
Omega_beta	1/day	0.43	n.e.
Omega_eta	1/(nanomole/liter)	1.76	0.34 (19.6%)
**Error model parameters**			
a		1.49	0.068 (4.54%)

n.e. = not estimated, shown for parameters whose values were fixed to the values estimated from the WT model
